# Diverse Circular DNA Viral Communities in Blood, Oral, and Fecal Samples of Captive Lemurs

**DOI:** 10.3390/v16071099

**Published:** 2024-07-08

**Authors:** Elise N. Paietta, Simona Kraberger, Michael C. Lund, Karla L. Vargas, Joy M. Custer, Erin Ehmke, Anne D. Yoder, Arvind Varsani

**Affiliations:** 1 Department of Biology, Duke University, Durham, NC 27708, USA; 2 The Biodesign Center for Fundamental and Applied Microbiomics, Center for Evolution and Medicine and School of Life Sciences, Arizona State University, Tempe, AZ 85287, USA; 3 Duke Lemur Center, Duke University, Durham, NC 27708, USA; 4 Structural Biology Research Unit, Department of Integrative Biomedical Sciences, University of Cape Town, Cape Town 7925, South Africa

**Keywords:** lemurs, *Anelloviridae*, *Cressdnaviricota*, *Microviridae*, *Inoviridae*, *Caudoviricetes*

## Abstract

Few studies have addressed viral diversity in lemurs despite their unique evolutionary history on the island of Madagascar and high risk of extinction. Further, while a large number of studies on animal viromes focus on fecal samples, understanding viral diversity across multiple sample types and seasons can reveal complex viral community structures within and across species. Groups of captive lemurs at the Duke Lemur Center (Durham, NC, USA), a conservation and research center, provide an opportunity to build foundational knowledge on lemur-associated viromes. We sampled individuals from seven lemur species, i.e., collared lemur (*Eulemur collaris*), crowned lemur (*Eulemur coronatus*), blue-eyed black lemur (*Eulemur flavifrons*), ring-tailed lemur (*Lemur catta*), Coquerel’s sifaka (*Propithecus coquereli*), black-and-white ruffed lemur (*Varecia variegata variegata*), and red ruffed lemur (*Varecia rubra*), across two lemur families (Lemuridae, Indriidae). Fecal, blood, and saliva samples were collected from Coquerel’s sifaka and black-and-white ruffed lemur individuals across two sampling seasons to diversify virome biogeography and temporal sampling. Using viral metagenomic workflows, the complete genomes of anelloviruses (*n* = 4), cressdnaviruses (*n* = 47), caudoviruses (*n* = 15), inoviruses (*n* = 34), and microviruses (*n* = 537) were determined from lemur blood, feces, and saliva. Many virus genomes, especially bacteriophages, identified in this study were present across multiple lemur species. Overall, the work presented here uses a viral metagenomics approach to investigate viral communities inhabiting the blood, oral cavity, and feces of healthy captive lemurs.

## 1. Introduction

Each distinct area of the body (e.g., oral and nasal cavities, circulating blood, gastrointestinal tract, cerebrospinal fluid, liver, skin) has the potential to harbor different and diverse viral communities. Further, distinct individuals, even within the same species, have been found to possess highly heterogeneous viromes [1]. Diverse biogeographical sampling across multiple individuals, therefore, charts a more refined landscape of a species’ virome. Studies on the human virome have attempted to address this, determining a foundation of prokaryote- and eukaryote-infecting viral diversity associated with humans based on diverse sampling [2]. However, even the human virome requires more extensive study as many viruses, especially bacteriophages, in recent metagenomic studies have little similarity to publicly available viral sequences [3]. In addition to humans, although to a lesser extent, viral metagenomic studies have diversified virome biogeography sampling in animals (e.g., laboratory rabbits [4], snakes [5], horses [6], rhesus macaques [7]), resulting in explorations of viral sequences across diverse microbe-, plant-, and vertebrate-infecting viral families in different sample types.

Blood, fecal, and oral viral communities can impact host health directly through pathogenic vertebrate-infecting viruses and indirectly through microbe-infecting viruses that influence bacterial, archaeal, or protozoan abundance and behavior. The blood virome is primarily studied to reveal vertebrate-infecting viruses (e.g., anelloviruses, retroviruses, flaviviruses) circulating throughout the blood to reach host tissues and organs [8]. Studies of the fecal virome have primarily yielded the genomes of expansive bacteriophage communities found to shift with factors such as age and diet [9,10]. Further, the oral virome comprises unique microbe-infecting communities inhabiting various niches within the oral cavity [2,3]. The oral and fecal environments also frequently harbor pathogenic (e.g., respiratory viruses or diarrheal disease-causing viruses) and nonpathogenic (e.g., anelloviruses) vertebrate-infecting viruses.

Despite their evolutionary relatedness to humans, viruses associated with non-human primates have been poorly investigated. Most studies have concentrated on the fecal viromes, as feces can be collected non-invasively [7,11,12,13,14,15]. The lemuriform primates, in particular, have been one of the most understudied primate lineages for virome research, with remarkably limited lemur-associated viral genomes available. Based on the NCBI Virus database [16], complete virus genomes available in GenBank from lemur samples include viruses only within the families *Adenoviridae*, *Anelloviridae*, *Circoviridae*, *Flaviviridae*, *Papillomaviridae*, *Parvoviridae*, *Picornaviridae*, *Retroviridae*, and *Smacoviridae*. The lemur papillomaviruses were identified as part of this project [17,18]. Lemurs make up ~20% of primate species, have a rich evolutionary history on the island of Madagascar, and are experiencing heightened anthropogenic-mediated change. Thus, characterizing viruses in lemurs is crucial both for understanding viral diversity in non-human primates and for endangered species conservation. 

Species-specific lemur behavior, diet, physiology, and environment are also likely to have shaped diverse viral communities inhabiting different lemur body sites. The species diversity of the lemuriform clade has evolved to take advantage of the diverse ecosystems of Madagascar—from tropical rainforests to dry deciduous forests to the spiny desert—through factors such as behavior, diet, morphology, and activity patterns [19,20,21]. The lemuriform clade is composed of five families (Lemuridae, Indriidae, Cheirogaleidae, Lepilemuridae, and Daubentoniidae) of 15 genera. As ~98% of lemurs are now threatened with extinction [22], some lemur species have extensive captive populations (e.g., *Lemur catta* and *Eulemur* in Florida (USA) at the Lemur Conservation Foundation, *Varecia rubra* in Germany at the Tierpark Berlin Zoo) that serve a vital role in Species Survival Plans managed by the Association of Zoos and Aquariums (AZA). 

The Duke Lemur Center (DLC) in Durham, North Carolina, USA is home to 14 lemur species across four lemur families (Lemuridae, Indriidae, Cheirogaleidae, and Daubentoniidae). Serving as a living laboratory for advancing interdisciplinary research, scholarship, and conservation, the DLC is the only place in the world where lemurs are readily available for comparative study together with associated biological samples, decades of medical records, and life history data. The diversity of the colony enables an expansive scope of science to be conducted and communicated. Here, we analyze samples from seven lemur species—collared lemur (*Eulemur collaris*), crowned lemur (*Eulemur coronatus*), blue-eyed black lemur (*Eulemur flavifrons*), ring-tailed lemur (*Lemur catta*), Coquerel’s sifaka (*Propithecus coquereli*), black-and-white ruffed lemur (*Varecia variegata variegata*), and red ruffed lemur (*Varecia rubra*)—across Lemuridae and Indriidae to build a foundation of viral diversity in captive lemurs. While only fecal samples were obtained from *Eulemur* and *Lemur* species, blood samples, saliva, and feces were collected from Coquerel’s sifaka and black-and-white ruffed lemurs to obtain diversified virome biogeography sampling. The primary aim of this study was to use viral metagenomics to start mapping vertebrate- and microbe-infecting viral communities inhabiting the blood, oral cavity, and feces of healthy captive lemurs. 

## 2. Materials and Methods

### 2.1. Sample Collection and Preparation

Samples were collected from captive lemurs at the DLC between August 2021 and September 2022. Black-and-white ruffed lemur individuals, Duke_23, Duke_24, and Duke_25, and Coquerel’s sifaka individuals, Duke_27, Duke_28, and Duke_30, were sampled for feces, blood, and saliva in August 2021 and March 2022. Fecal samples were collected from collared lemur (*n* = 1), crowned lemur (*n* = 1), blue-eyed black lemur (*n* = 1), ring-tailed lemur (*n* = 1), Coquerel’s sifaka (*n* = 8 samples, 4 individuals), and black-and-white ruffed lemur (*n* = 10 samples, 4 individuals) individuals immediately after defecation and frozen at −80 °C until processing. To prepare for nucleic acid extraction, fecal samples were thawed, homogenized with SM buffer (0.1 M NaCl, 50 mM Tris-HCl [pH 7.4]), and centrifuged for 10 min, and the supernatant was filtered through 0.45 µm and 0.2 µm syringe filters sequentially. Oral swab samples were collected from black-and-white ruffed lemurs (*n* = 7 samples, 4 individuals), red ruffed lemurs (*n* = 4 samples, 4 individuals), and Coquerel’s sifakas (*n* = 6 samples, 4 individuals). Oral samples were collected by allowing the lemurs to chew on a SalivaBio Children’s Swab (Salimetrics, Carlsbad, CA, USA). The saturated swabs were then placed into a SalivaBio Swab Storage Tube (Salimetrics, Carlsbad, CA, USA) and centrifuged to collect the saliva. Oral samples were stored at −80 °C until processing. To prepare for nucleic acid extraction, SM buffer was added to the spun saliva up to a final volume of 400 µL and homogenized prior to extraction. For blood draws from black-and-white ruffed lemurs (*n* = 6 samples, 3 individuals) and Coquerel’s sifakas (*n* = 7 samples, 4 individuals), 0.5–1 mL of whole-blood samples was collected from the femoral vein of each lemur. Blood samples were stored at −80 °C until processing. All lemurs sampled for blood were above 2 years of age and in good health at the time of sampling. As only larger-bodied lemurs were sampled, all blood draws were well within the safe limits determined by IACUC. The blood, fecal, and saliva samples were collected under IACUC #A161-21-08. Singular fecal samples from collared lemur, crowned lemur, blue-eyed black lemur, and ring-tailed lemur individuals were collected under IACUC #A109-20-05. 

### 2.2. Extraction, Library Preparation, and Sequencing

Following sample preparation, 200 µL of filtrate, homogenate, or blood sample was used for viral nucleic acid extraction using the High Pure Viral Nucleic Acid Kit (Roche Diagnostics, Indianapolis, IN, USA). DNA extracts were amplified using the Illustra Templiphi rolling circle amplification kit (GE Healthcare, Chicago, IL, USA) to target circular DNA viruses. Libraries were generated using the Illumina DNA Prep Kit and sequenced using the Illumina NovaSeq 6000 at the Duke Center for Genomic and Computational Biology (Durham, NC, USA), except for those from red ruffed lemur saliva, which were sequenced on an Illumina HiSeq 2500 at Psomagen Inc. (Rockville, MD, USA). 

### 2.3. Virus De Novo Assembly and Genome Identification

Paired-end reads (2 × 150 bp) were trimmed using Trimmomatic-0.39 [23] and de novo assembled with MEGAHITv.1.2.9 [24]. Circular contigs were identified based on terminal redundancy and contigs >1000 nts were analyzed for viral-like sequences using Diamond [25] BLASTx against a local viral RefSeq protein sequence database (Release 220). Viral genomes, except bacteriophages, were annotated using CenoteTaker2 [26]. Large phage, microvirus, and inovirus genomes were annotated with VIBRANT [27]. All annotations were manually checked. Pairwise identity calculations were determined with the Sequence Demarcation Tool (SDT) v1.2 [28] for anelloviruses and cressdnaviruses, and virus intergenomic similarities were computed with VIRIDIC [29] for the large phages and inoviruses.

### 2.4. Distribution of Virus Genomes across Samples

Virus operational taxonomic units (vOTUs), comprised of virus genomes with greater than 98% identity, were clustered using CD-Hit [30]. Using BBMap [31], Illumina sequencing raw reads were then mapped to a representative genome from each vOTU to determine the distribution of viruses across all samples.

### 2.5. Phylogenetic Analyses

#### 2.5.1. Anelloviruses

Genome sequences of viruses in the *Anelloviridae* family were downloaded from GenBank. From the available GenBank sequences and anelloviruses identified in this study, *orf1* genes were extracted, translated, and aligned using MAFFT v.7.113 [32]. The alignment was used to infer a maximum likelihood phylogenetic tree using PhyML 3.0 [33]. The best-fit amino acid substitution model, VT + G + F, was determined using ProtTest 3 [34]. The phylogenetic tree was rooted with sequences of anelloviruses in the genus *Gyrovirus*. Genus-level branches were collapsed and the tree was visualized with iTOL v6 [35]. 

#### 2.5.2. Cressdnaviruses

Replication-associated protein (Rep) sequences were extracted from the cressdnavirus genomes identified in this study and from a dataset of representative cressdnavirus genomes in the families *Bacillidnaviridae*, *Circoviridae*, *Geminiviridae*, *Genomoviridae*, *Metaxyviridae*, *Nanoviridae*, *Naryaviridae*, *Nenyaviridae*, *Redondoviridae*, *Smacoviridae*, and *Vilyaviridae*, as well as groups CRESSV1-6 [36] and those of *Alphasatellitidae*. To determine the family-level assignment of the cressdnaviruses, a sequence similarity network (SSN) was generated with the extracted Rep amino acid sequences using EFI-EST [37] with a sequence similarity score of 60, a score previously shown to reveal cressdnavirus family-level groupings [38,39,40,41,42,43,44,45]. The SSN was then visualized with Cytoscape v3.8.2 [46].

The Rep sequences forming clusters with cressdnaviruses from this study and established viral cressdnavirus families or clusters (CRESSV1-6, UC1-7) were extracted and subsequently aligned with MAFFT v7.113 [32]. The alignment was trimmed with TrimAL (0.2 gap threshold) [47]. The trimmed alignment of Rep sequences was then used to infer a maximum likelihood phylogenetic tree with IQTree 2 [48] using the best-fit substitution model, Q.pfam + F + G4, which was determined using ProtTest 3 [34]. This phylogeny was visualized and annotated with iTOL v6 [35]. 

From the SSN, for each cluster containing Rep sequences of the cressdnaviruses identified in this study, we aligned the Rep amino acid sequences using MAFFT v7.113 [32]. Alignments were then used to infer maximum likelihood phylogenetic trees using PhyML 3.0 [33]. Best-fit amino acid substitution models were determined using ProtTest 3 [34] (LG + I + G + F for CRESSV1, LG + I + G for CRESSV6, RtREV + I + G + F for UC1, RtREV + I + G + F for UC2, RtREV + I + G + F for UC3/UC4, LG + G + F for UC5, LG + G + F for UC6, LG + I + G + F for gemycircularviruses, LG + I + G + F for gemykibiviruses, LG + I + G + F for gemykrogviruses, LG + I + G for curtoviruses, LG + I + G + F for smacoviruses, and RtREV + I + G + F for vilyaviruses). Branches with <0.8 aLRT support were collapsed with TreeGraph2 [49]. 

#### 2.5.3. Microviruses

Major capsid protein (MCP) sequences were extracted from microvirus genomes identified in this study and a dataset of publicly available complete microvirus genomes from GenBank. MCP sequences were translated and aligned with MAFFT v7.113 [32]. The alignment was trimmed with TrimAL [47] (0.2 gap threshold) and used to infer a maximum likelihood phylogenetic tree using IQTree 2 [48] with LG + F + G4 as the best-fit amino acid substitution as determined using ProtTest3 [34]. The tree was visualized using iTOL v6 [35]. Microvirus host taxonomy to the genus rank was predicted using iPHoP [50].

#### 2.5.4. Large Bacteriophages and Inoviruses

For caudoviruses, a proteomic tree of dsDNA bacteriophages was generated with ViPTree server version 4.0 [51]. For inoviruses, complete genomes identified in this study, along with a custom database of inoviruses, were used to infer a proteomic tree using ViPTree server version 4.0. For both caudovirus and inovirus clades, within-clade intergenomic distances were calculated using VIRIDIC [29]. Inovirus and caudovirus bacterial host taxonomy to the genus rank was predicted using iPHoP [50].

## 3. Results and Discussion

Feces from collared lemur, crowned lemur, blue-eyed black lemur, Coquerel’s sifakas, and black-and-white ruffed lemurs, saliva from Coquerel’s sifakas, red ruffed lemurs, and black-and-white ruffed lemurs, and blood samples from Coquerel’s sifakas and black-and-white ruffed lemurs were analyzed using viral metagenomic workflows optimized to identify circular DNA viruses. 

**Figure 1:**
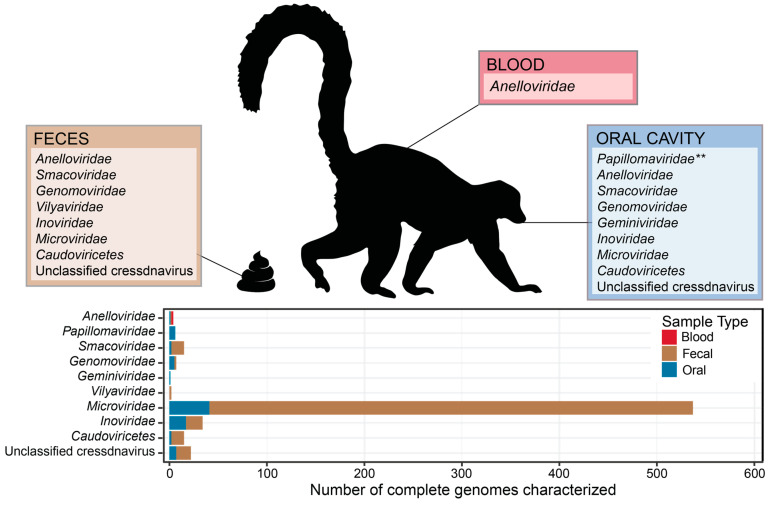
Viral families (and viral order for *Caudoviricetes*) characterized from blood, fecal, and oral samples from lemur individuals at the Duke Lemur Center (Durham, NC, USA). ** Lemur papillomaviruses described from Duke Lemur Center samples have been previously published [17,18]. Lemur image from PhyloPic.

**Figure 2:**
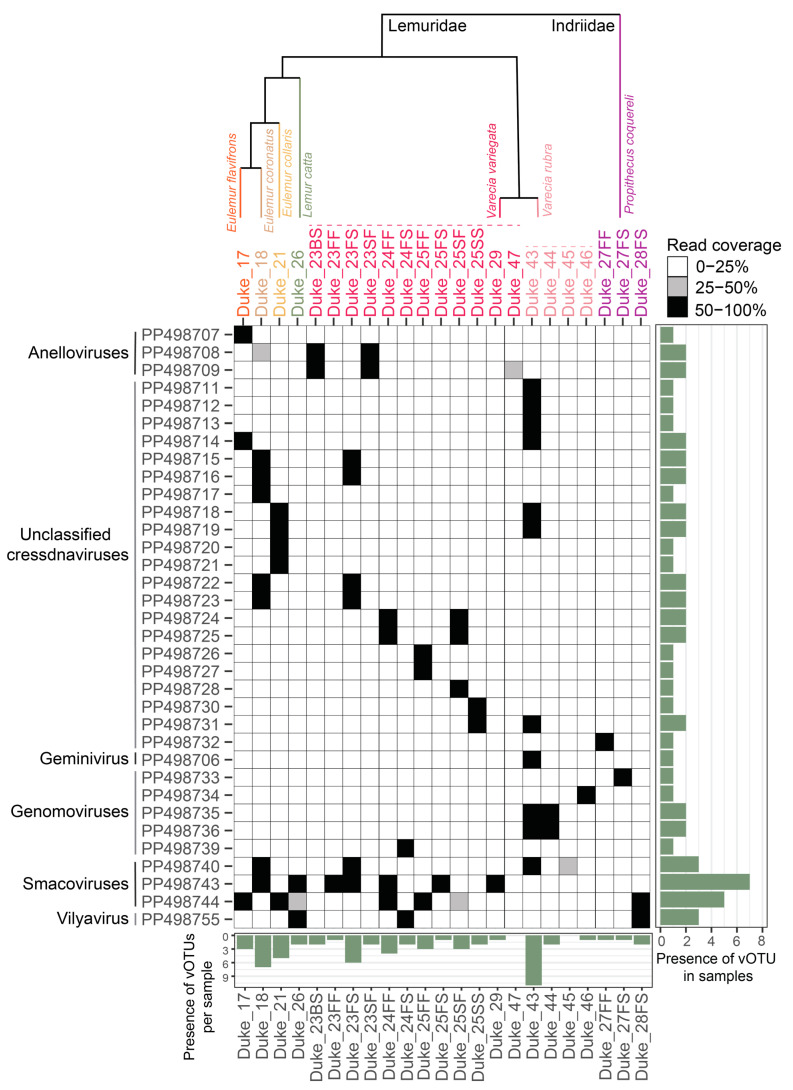
A genome coverage plot illustrating the presence of binned vOTUs identified in this study of eukaryote-infecting viruses and those in the family *Smacoviridae* across all samples. Black-colored squares represent 50–100% genome coverage, gray-colored squares represent 25–50% genome coverage, and white-colored squares represent 0–25% genome coverage. If read coverage is >50%, this represents a high-confidence proxy of vOTU presence in a sample. The bar graph to the right of the plot depicts the number of samples in which a particular vOTU is present with high confidence (>50% coverage). The bar plot below depicts the number of vOTUs present in a particular sample. The phylogenetic relationship between lemur species sampled is depicted above the sample names (note that there are >100 lemur species, so the relationship between species sampled is approximately shown).

**Figure 3:**
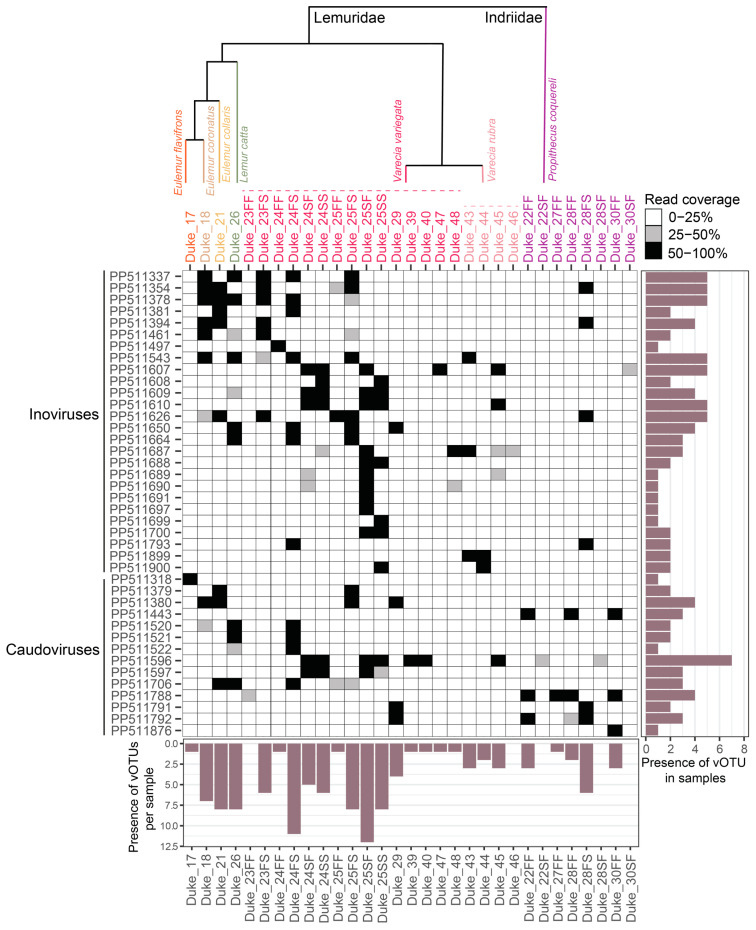
A genome coverage plot illustrating the presence of inovirus and caudovirus vOTUs identified in this study across all samples. Black-colored squares represent 50–100% genome coverage, gray-colored squares represent 25–50% genome coverage, and white-colored squares represent 0–25% genome coverage. If read coverage is >50%, this represents a high-confidence proxy of vOTU presence in a sample. The bar graph to the right of the plot depicts the number of samples in which a particular vOTU is present. The bar graph below the heatmap depicts the number of vOTUs present in a particular sample with high confidence (>50% coverage). The phylogenetic relationship between lemur species sampled is depicted above the sample names (note that there are >100 lemur species, so the relationship between species sampled is approximately shown).

From these samples, 637 complete virus genomes were de novo assembled representing viruses in the taxa *Anelloviridae* (4 complete genomes), *Cressdnaviricota* (47 complete genomes), *Microviridae* (537 complete genomes), *Inoviridae* (34 complete genomes), and *Caudoviricetes* (15 complete genomes; Table S1). Different sample types contained viruses across diverse families (Figure 1; Table S1). We identified 269 distinct vOTUs which have been named dulem virus (DlemV) 1 through 269. Circular DNA virus composition differed widely across individuals and species (Figure 2 and Figure 3). While virus genomes in the *Anelloviridae* family appeared to be species-specific (Figure 2), many cressdnaviruses and bacteriophages were present across multiple individuals and species (Figure 2 and Figure 3). The work presented here aims to begin mapping the landscape of the DNA viromes of healthy captive lemurs. 

### 3.1. Novel Anelloviruses in Blood, Oral, and Fecal Samples

The *Anelloviridae* family consists of viruses classified into 30 genera. Anelloviruses have small, circular, single-stranded DNA genomes around 1.6–3.9 kb in length with 3–4 open reading frames (ORFs) and a conserved noncoding GC-rich region [52,53]. Anelloviruses infect eukaryotes with no known negative impacts on the health of their hosts [54,55]. *Orf1*, encoding a capsid protein [56], is highly conserved across anelloviruses and serves as the basis for determining new species (69% species demarcation threshold) [52,57]. Host species-specific anelloviruses are consistently found in tissue, blood, fecal, nasal, and oral viromes of mammals, avians, and even invertebrates (likely due to a recent blood meal) [52,58,59,60,61,62,63]. Anelloviruses have been detected across most primate lineages including great apes, New World monkeys, Old World monkeys, and a lemuriform [53,63,64]. 

Despite the extensive evolutionary diversity of lemurs within the primate order, only a single anellovirus genome has been published for lemuriform primates. Torque teno indri virus 1 (TTIV1; MF187212) is a member of the species *Chitorquevirus indri1*, identified in a blood sample from a free-living *Indri indri* [53]. The TTIV1 genome is 2572 nt in length and highly divergent, sharing 27% ORF1 identity with that of anellovirus procyon3206 (BK066324) from a raccoon [65]. As anelloviruses appear to be ubiquitous and highly prevalent across primates, diverse species-specific anelloviruses are likely to exist across the diverse lemuriform clade [58].

**Figure 4:**
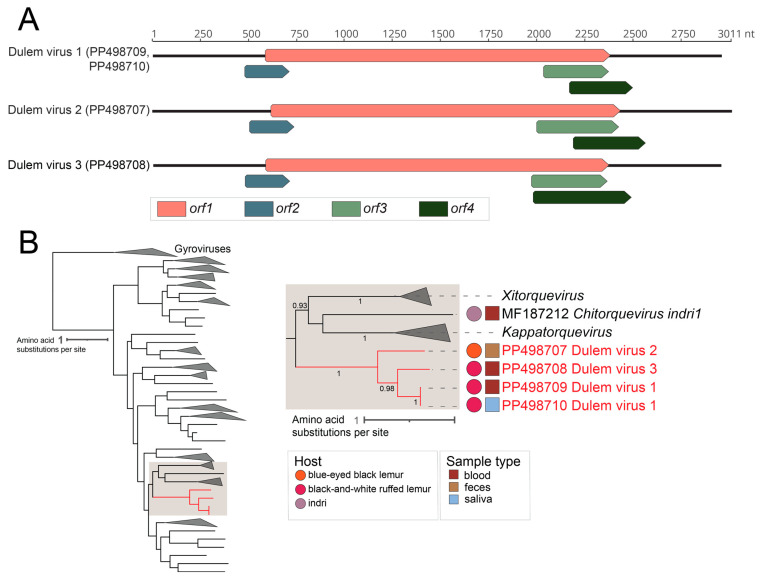
(**A**) Linearized genome organization of the four anellovirus genomes identified in this study. (**B**) A maximum likelihood phylogenetic tree of the ORF1 amino acid sequences of representative members of the *Anelloviridae* family. A segment of the phylogenetic tree containing anelloviruses of interest is shown in detail on the right. For lemur anelloviruses, their host is depicted by a colored circle, while sample type is denoted by a colored square. Virus sequences identified in this study are in red font, forming their own cluster within the *Anelloviridae* family, separate from the only other known lemur anellovirus *Chitorquevirus* indri1 identified by Amatya et al. (2017) [53].

In this study, four complete anellovirus genomes (2952-3011 nts; Figure 4A) were identified in blue-eyed black lemur (Duke_17) feces (DlemV2, PP498707), black-and-white ruffed lemur (Duke_23) blood (DlemV1, PP498709; DlemV3, PP498708), and black-and-white ruffed lemur (Duke_23) saliva (DlemV1, PP498710) (Figure 4A). The *orf1* nucleotide sequences of these anelloviruses from black-and-white ruffed lemurs and a blue-eyed black lemur in the Lemuridae family share 59.1–99.9% identity and are phylogenetically closely related compared to the *orf1* of the anellovirus (MF187212) from an indri in the Indriidae family (Figure 4B).

DlemV1 (PP498709 and PP498710) genomes are 2955 nt in length and share 99.9% *orf1* nt identity. The *orf1*s of DlemV1 (PP498709 and PP498710) share ~68.8% and 59.1% nt identity with those of DlemV3 (PP498708) and DlemV2 (PP498707), respectively. The ORF1 of DlemV2 shares ~61% amino acid pairwise identity with that of DlemV3. Based on the 69% species demarcation threshold, DlemV1 and DlemV3 represent two new species from black-and-white ruffed lemurs and DlemV2 represents a new species from a blue-eyed black lemur. As black-and-white ruffed lemur anelloviruses share more similarity with one another than with the blue-eyed black lemur anellovirus and both show low similarity with *Chitorquevirus indri1*, this study suggests a marked host species-level anellovirus speciation. The two DlemV1 variants were present across two sample types, blood and saliva, of the same black-and-white ruffed lemur individual, Duke_23, and this animal also had a coinfection with DlemV3 in the blood. These anelloviruses from black-and-white ruffed lemurs and a blue-eyed black lemur represent three new species and are members of a putative new genus in the family *Anelloviridae*. 

### 3.2. Diverse Cressdnaviruses in Fecal and Oral Samples

Viruses in the twelve families of *Cressdnaviricota* have been found to infect primarily eukaryotic hosts including associations with animals (*Circoviridae* [66]), plants (*Geminiviridae* [67], *Metaxyviridae*, *Nanoviridae* [68], *Amesuviridae* [69,70]), fungi (*Genomoviridae* [71]), protists (*Vilyaviridae*, *Naryaviridae*, *Nenyaviridae* [72], *Redondoviridae* [73]), and diatoms (*Bacilladnaviridae*), in addition to fecal archaea (*Smacoviridae* [74]). As the *Cressdnaviricota* phylum is rapidly expanding, many cressdnaviruses cannot yet be assigned to classified families. Cressdnaviruses, in general, have small circular, single-stranded DNA genomes encoding a capsid protein (CP) and replication-associated protein (Rep). All cressdnaviruses have an HUH endonuclease domain [75] and Superfamily 3 helicase domain [76] in their Reps. In addition, Reps of viruses in the families *Geminiviridae* and *Genomoviridae* have the gemini Rep Sequence (GRS) domain [77]. 

**Figure 5:**
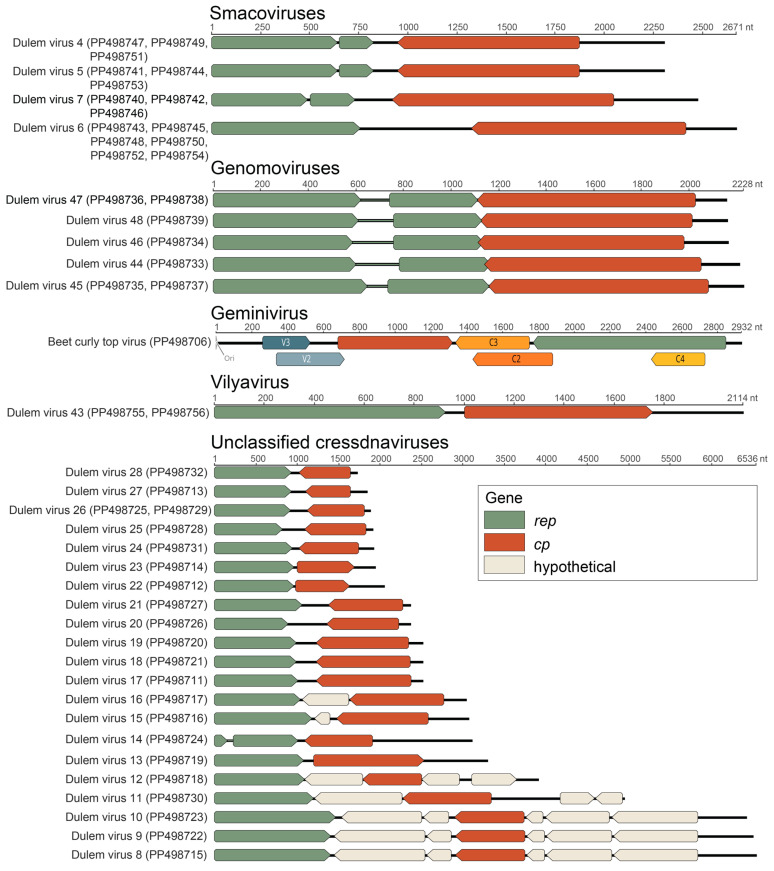
Linearized representation of the genome organization of the cressdnaviruses identified in this study. Accession numbers of genomes displayed in the same row share >98% nucleotide identity.

**Figure 6:**
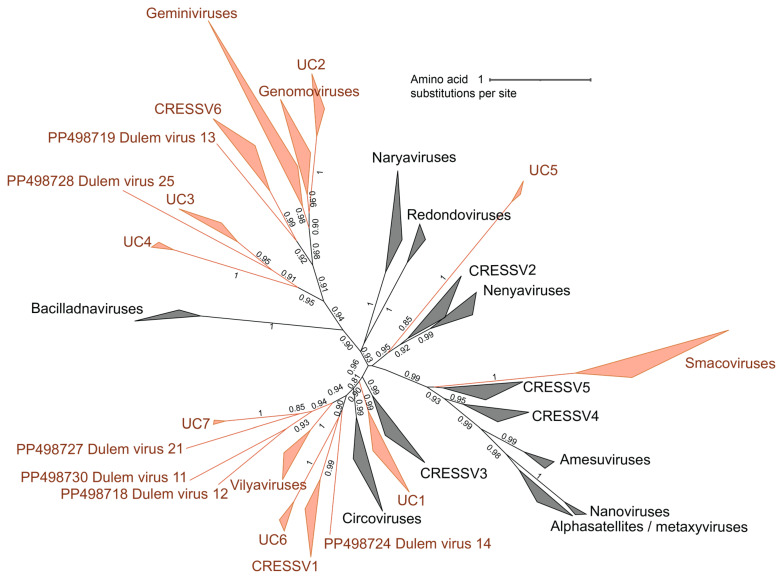
A maximum likelihood phylogenetic tree of the Rep sequences of viruses in the phylum *Cressdnaviricota*, separated into family-level clustering. Family-level clusters, which include virus genomes characterized in this study, are in orange. UC1-7 represent unclassified family-level clusters. Unclassified cressdnavirus genomes that do not fall into family-level clusters are singletons.

Forty-seven cressdnavirus genomes (Figure 5) were identified from blue-eyed black lemur, collared lemur, crowned lemur, ring-tailed lemur, red ruffed lemur, and black-and-white ruffed lemur fecal and oral samples. Based on Rep sequence analysis, 15 genomes are part of the family *Smacoviridae*, 7 are part of the family *Genomoviridae*, 1 is within the family *Geminiviridae*, 2 are part of the family *Vilyaviridae,* 16 form clusters with various unclassified cressdnaviruses (UC1-7), and 6 are singletons (Figure 6). All cressdnavirus genomes in this study encode capsid (CP) and replication-associated (Rep) proteins (Figure 5). Motifs in the cressdnavirus Reps identified in this study are summarized in Table S2. The HUH endonuclease Motifs I, II, and III and the SF3 helicase Walker A, Walker B, and Motif C were identified in all cressdnavirus genomes. Arg finger was identified in most cressdnavirus genomes, and the GRS domain was identified for the genomoviruses and geminivirus characterized in this study.

#### 3.2.1. Smacoviruses

Smacoviruses have primarily been identified from various animal fecal samples. Smacovirus genomes are ~2.3–3 kb in length and contain two genes encoding for a CP and Rep. Viruses within the *Smacoviridae* family are divided into 12 genera. The genus *Porprismacovirus* contains smacoviruses associated with pig and primate samples [78]. Fecal archaea *Candidatus Methanomassiliicoccus intestinalis* and *Candidatus Methanomethylophilus*, identified through analysis of CRISPR spacer sequences, remain the leading candidate hosts of viruses in *Smacoviridae* [74,79]. In non-human primates, smacoviruses have been found to be associated with chimpanzees—where they were first detected—gorillas, howler monkeys, and ring-tailed lemurs [11,15,80,81]. Three lemur-associated virus genomes in the *Porprismacovirus* genus, representing two smacovirus species, had been previously characterized in the feces of captive ring-tailed lemurs [15]. 

**Figure 7:**
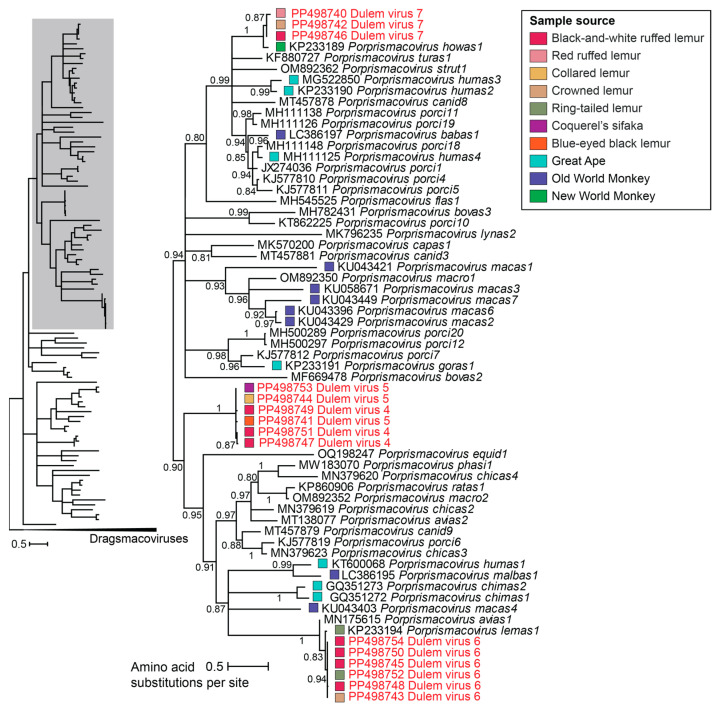
A maximum likelihood phylogenetic tree of Rep amino acid sequences of viruses in the *Porprismacovirus* genus rooted with selected dragsmacovirus sequences. A segment of the phylogenetic tree containing smacoviruses of interest is displayed. Colored boxes denote a smacovirus identified from a non-human primate sample. For classified viruses, assigned smacovirus species names are presented next to virus accession numbers. Viruses identified in this study are depicted in red font.

Smacovirus genomes identified in this study were determined from collared lemur (*n* = 1), crowned lemur (*n* = 2), blue-eyed black lemur (*n* = 1), ring-tailed lemur (*n* = 1), Coquerel’s sifaka (*n* = 1), and black-and-white ruffed lemur (*n* = 7) feces, and black-and-white ruffed lemur (*n* = 1) and red ruffed lemur (*n* = 1) saliva. The smacovirus genomes identified in this study form three clusters within the *Porprismacovirus* genus. For one distinct cluster, Reps in smacovirus genomes identified from Coquerel’s sifaka (DlemV5, PP498753), collared lemur (DlemV5, PP498744), black-and-white ruffed lemur (DlemV4, PP498749, PP498747), and crowned lemur (DlemV5, PP498741) feces and the saliva of a black-and-white ruffed lemur (DlemV4, PP498751) share 97–99% amino acid identity, thus showing that the same smacovirus species can be found across diverse Lemuridae and Indriidae sample sources (Figure 7). Further, DlemV4 shares >98% genome similarity with Dumus virus 2 isolate Duke_15_113 (PP473146) from house mouse (*Mus musculus*) feces collected from mice entering enclosure areas at the Duke Lemur Center as part of this overall project. The genomes of DlemV4 and DlemV5 share >97% similarity and thus represent a new smacovirus species based on the 77% genome-wide species demarcation threshold for *Smacoviridae* [78].

Smacoviruses from black-and-white ruffed lemur (DlemV6; PP498754, PP498750, PP498745, PP498748), ring-tailed lemur (DlemV6; PP498752), and crowned lemur (DlemV6; PP498743) feces cluster with lemur-associated porprismacovirus 1 isolate SF5 (KP233194) of the species *Porprismacovirus lemas1* from a ring-tailed lemur at the San Francisco Zoo [15]. The Reps of DlemV6 share >99% amino acid identity with one another and ~98% amino acid identity with lemur-associated porprismacovirus 1 isolate SF5 (Figure 7). DlemV6 genomes share ~92% similarity with lemur-associated porprismacovirus 1 isolate SF5 (KP233194; species *Porprismacovirus lemas1*). As this falls above the 77% species demarcation threshold, DlemV6 is a member of the species *Porprismacovirus lemas1*. Clustering with howler monkey-associated porprismacovirus 1 isolate SF1 (KP233189) of the species *Porprismacovirus howas1* from a howler monkey at the San Francisco Zoo (CA, USA), Reps from DlemV7 (PP498742, PP498746, PP498740) determined from crowned lemur and black-and-white ruffed lemur feces and red ruffed lemur saliva share ~98–100% amino acid identity with one another and share 90–94% amino acid identity with *Porprismacovirus howas1* (Figure 7). DlemV7 genomes share ~69% similarity with howler monkey-associated porprismacovirus 1 isolate SF1 (KP233189; *Porprismacovirus howas1*) and >99% similarity with one another. Consequently, DlemV7 represents a new smacovirus species. 

This study identified the same smacovirus species across the source samples of four lemur species spanning three genera (*Eulemur*, *Propithecus*, *Varecia*) and two families (Lemuridae, Indriidae). One smacovirus species was found in the feces and saliva of black-and-white ruffed lemurs in addition to feces from a house mouse entering lemur enclosure areas at the Duke Lemur Center. Further, the smacovirus genomes clustered with *Porprismacovirus lemas1* or *Porprismacovirus howas1* emphasize that the host of smacoviruses, most likely methanogenic fecal archaea [79], is prevalent in the feces of non-human primates. DlemV7’s high similarity with *Porprismacovirus howas1* from howler monkeys shows that the host of smacoviruses is likely not species-specific or even primate suborder-specific. Additionally, although smacoviruses were identified in the saliva of a black-and-white ruffed lemur and red ruffed lemur, this finding is potentially the result of coprophagy or geophagy [82]. Ruffed lemurs have been documented to descend to the ground and eat soil in Betampona Reserve [82] and Ranomafana National Park [83] in Madagascar. Geophagy in non-human primates has been suggested to supplement minerals in the diet [84] or to aid in neutralizing tannins [85]. Coprophagy has been suggested to be a nutritional supplement, especially in more stressful environments, for lemurs [86]. The ruffed lemur oral samples with smacoviruses were collected during a time period in which the lemur individuals were free-ranging in the forest. Ruffed lemur geophagy has not been seen in recorded observations, studies, or husbandry anecdotally at the DLC. However, ruffed lemurs have been regularly observed to participate in coprophagy at the DLC [86]. The smacoviruses, therefore, may be the result of coprophagy, and if this is the case, it could allow us to use viruses to connect to lemur behavior. 

#### 3.2.2. Genomoviruses

Genomoviruses have been detected in diverse sample sources including fungi [87], plants [88,89,90], vertebrates (e.g., humans [44,60,91], non-human primates [92,93], bats [94,95], pangolins [96], rodents [44], whales [97]), and invertebrates [98]. Fungi are the probable host of genomoviruses as at least two genomovirus species (*Gemycircularvirus sclero1* and *Gemytripvirus fugra1*) have been confirmed to infect fungi [99,100]. Viruses in the *Genomoviridae* family are currently divided into 10 genera containing over 230 species, with 78% genome-wide identity as the species demarcation threshold [71]. Genomovirus genomes are ~1.8–2.4 kb in size.

**Figure 8:**
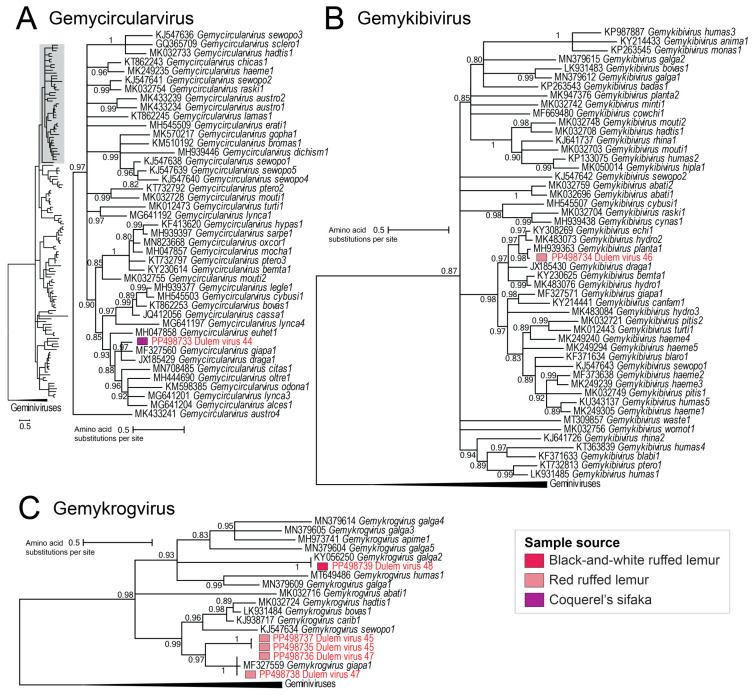
(**A**) A maximum likelihood phylogenetic tree of Rep sequences of genomoviruses in the *Gemycircularvirus* genus rooted with selected Rep sequences from the *Geminiviridae* family. (**B**) A maximum likelihood phylogenetic tree of Rep sequences of genomoviruses in the *Gemykibivirus* genus rooted with selected Rep sequences from the *Geminiviridae* family. (**C**) A maximum likelihood phylogenetic tree of Rep sequences of genomoviruses in the *Gemykrogvirus* genus rooted with selected Rep sequences from the *Geminiviridae* family. Assigned genomovirus species names are presented next to virus accession numbers. Virus genomes identified in this study are depicted in red font.

Genomoviruses in the genera *Gemycircularvirus*, *Gemykibivirus*, and *Gemykrogvirus* were characterized in this study from black-and-white ruffed lemur and Coquerel’s sifaka feces and red ruffed lemur saliva (Figure 8). DlemV44 (PP498733), within the *Gemycircularvirus* genus, shares 64–70% genome sequence identity with the giant panda-associated gemycircularvirus strain gpge003 (MF327560) [61] in the species *Gemycircularvirus giapa1*, and dragonfly-associated circular virus 2 isolate FL2-5X-2010 (JX185429) [101] in the species *Gemycircularvirus draga1*. As this falls below the 78% genome-wide species demarcation threshold [71] for genomoviruses, DlemV44 from Coquerel’s sifaka feces represents a novel gemycircularvirus species (Figure 8A). 

Within the *Gemykibivirus* genus, DlemV46 (PP498734) from red ruffed lemur saliva clusters with thrips-associated genomovirus 3 (KY308269; species *Gemykibivirus echi1*) [102], capybara genomovirus 2 (MK483073; species *Gemykibivirus hydro2*) [40], and plant-associated genomovirus 2 (MH939363; species *Gemykibivirus planta1*) [103]. As DlemV46 shares 78.1% sequence identity with plant-associated genomovirus 2 (MH939363), DlemV46 is part of the species *Gemykibivirus plantas1* (Figure 8B). 

In the *Gemykrogvirus* genus, DlemV48 (PP498739) from black-and-white ruffed lemur feces, is a member of a known species *Gemykrogvirus galga2* as it shares 97% genome identity with the chicken stool-associated gemycircularvirus strain RS/BR/2015 (KY056250) [104] (Figure 8C). DlemV45 (PP498735, PP498737) and DlemV47 (PP498736, PP498738), from two red ruffed lemur individuals’ saliva, form a distinct cluster with the giant panda-associated gemycircularvirus strain gpge002 (MF327559) [61] in the species *Gemykrogvirus giapa1*. DlemV45 genomes represent a new gemykrogvirus species as they share <65% with all other gemykrogviruses and >99% with one another (Figure 8C). On the other hand, DlemV47 shares >97% genome similarity with giant panda-associated gemycircularvirus (MF327559), making it a member of the species *Gemykrogvirus giapa1*.

The genomovirus genomes presented here, from lemur fecal and oral samples, represent two novel and three known genomovirus species across three *Genomoviridae* genera, *Gemycircularvirus*, *Gemykibivirus,* and *Gemykrogvirus*. These viruses likely infect fungi that are part of the microbial flora of the lemurs or their diet. 

#### 3.2.3. Geminivirus

Plant viruses, from families such as *Geminiviridae*, *Nanoviridae*, *Partitiviridae*, *Tobamoviridae*, and *Virgaviridae*, have frequently been identified in mammal fecal and oral viromes (e.g., human [105,106], non-human primate [92,107], domestic animal [108,109], rodent [110], bat [111]) likely due to recent consumption of infected plant material. The *Geminiviridae* family is composed of 14 genera. These plant-infecting single-stranded DNA viruses are transmitted between plants (cultivated and non-cultivated) by insect vectors, including aphids, leafhoppers, treehoppers, and whiteflies [67]. Geminiviruses cause numerous major crop diseases, making them a leading agricultural threat, especially for tropical and subtropical regions [67,112]. Geminiviruses have 2.5–5.2 kb circular genomes with either mono- or bipartite organization [67]. All geminivirus genomes encode for coat (CP), movement (MP), and Rep proteins; however, gene organization varies across viruses in different *Geminiviridae* genera. 

**Figure 9:**
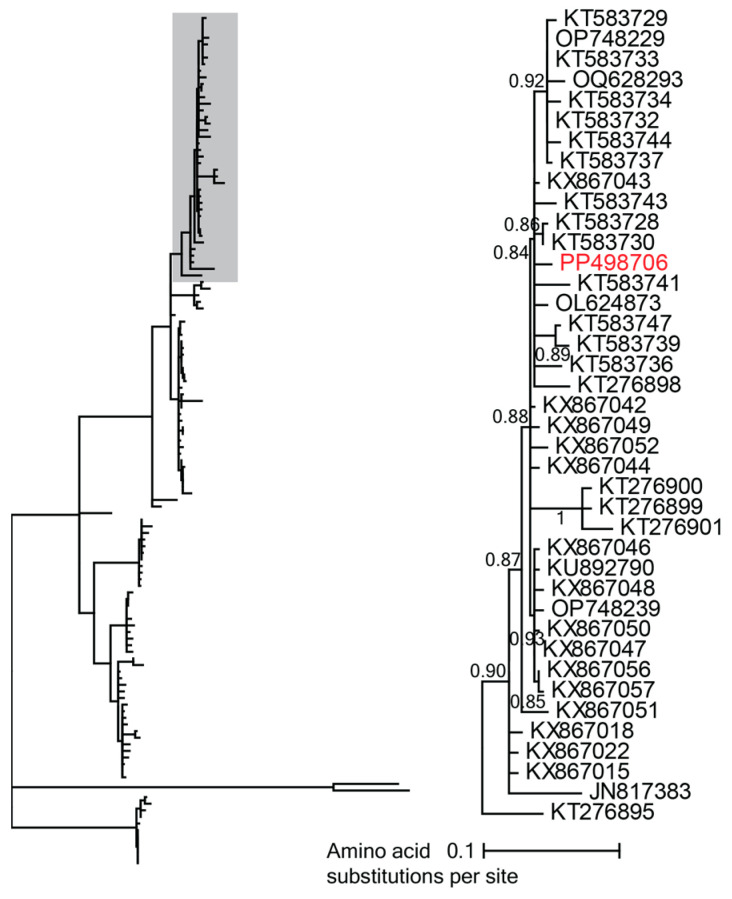
A maximum likelihood phylogenetic tree of the Rep sequences of viruses in the genus *Curtovirus* rooted with representative sequences of mastreviruses. A segment of the phylogenetic tree containing beet curly top virus isolates is shown in detail. The beet curly top virus isolate identified in this study from red ruffed lemur saliva is depicted in red.

In this study, one geminivirus genome, beet curly top virus (PP498706), 2932 nt in length, was characterized from red ruffed lemur saliva. This geminivirus is a beet curly top virus within the *Curtovirus* genus (Figure 9). Beet curly top virus (PP498706) shares 98.9% genome-wide pairwise identity with beet curly top virus isolates from tomatoes in California, USA (KT583728, KT583730). Beet curly top virus is a pathogenic plant virus widely impacting fruit and vegetable production, causing extensive economic losses [113]. At the DLC, ruffed lemurs consume a variety of cultivated fruits and vegetables along with forage from the DLC’s natural habitat enclosures. Beet curly top virus identified from a red ruffed lemur oral swab was likely infecting fruits or vegetables being consumed by the lemur.

#### 3.2.4. Vilyaviruses

The *Vilyaviridae* family currently contains viruses classified into 12 genera. Vilyavirus genomes are ~2 kb in length [72]. Vilyaviruses are predicted to infect protozoan parasites in the genus *Giardia* due to the identification of Rep sequences in *Giardia duodenalis* [114]. *G. duodenalis* and *Giardia* spp. have been found to be prevalent in both captive [115,116,117] and wild [116] lemur populations. Although *G. duodenalis* infection appears to be asymptomatic, especially in ring-tailed lemurs, *Giardia* is known to cause giardiasis symptoms (e.g., diarrhea, vomiting, cramps, weight loss, failure to thrive) in humans [118] and non-human primates [119,120]. *G. duodenalis* is considered a zoonotic pathogen as it causes giardiasis in humans and animals, presenting a risk to caretakers at captive animal facilities [121]. 

**Figure 10:**
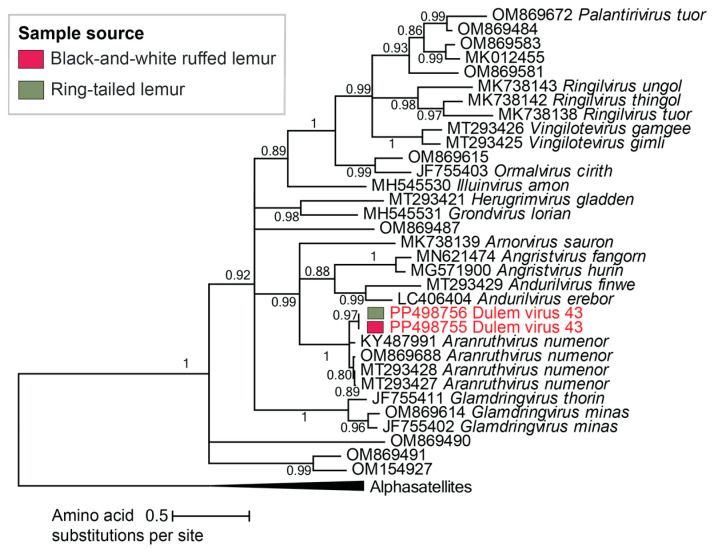
A maximum likelihood phylogenetic tree of the Rep sequences of viruses in the family *Vilyaviridae* rooted with representative sequences of alphasatellites. Assigned vilyavirus species names are presented next to virus accession numbers. Virus genomes identified in this study are depicted in red font.

Two vilyavirus genomes sharing 100% genome-wide pairwise identity were identified in ring-tailed lemur and black-and-white ruffed lemur fecal samples. These vilyavirus genomes are 2114 nt in length with CP and Rep proteins characteristic of cressdnavirus genomes. DlemV43 (PP498756 and PP498755) shares 81% genome-wide pairwise sequence identity with dipodfec virus RodF1_123 (OM869688), a vilyavirus genome characterized from Merriam’s kangaroo rat feces in Arizona (USA) [44] and a part of the species *Aranruthvirus numenor*. As 81% falls above the 78% genome-wide species demarcation threshold [72] for vilyaviruses, DlemV43 is a member of *Aranruthvirus numenor* (Figure 10). These findings suggest potential *Giardia* infection in one black-and-white ruffed lemur and one ring-tailed lemur at the DLC and emphasize our ability to use viruses to look for non-viral pathogens.

#### 3.2.5. Unclassified Cressdnaviruses

Many recently identified cressdnaviruses representing new species and putative families remain unclassified within the *Cressdnaviricota* phylum. In this study, 22 unclassified cressdnavirus genomes were characterized from six lemur species’ fecal and oral samples across three lemur genera (*Eulemur*, *Propithecus*, *Varecia*). Unclassified cressdnavirus genomes were determined from collared lemur feces (PP498718-PP498721), Coquerel’s sifaka feces (PP498732), crowned lemur feces (PP498715-PP498717), blue-eyed black lemur feces (PP498714), red ruffed lemur saliva (PP498711-PP498713), black-and-white ruffed lemur feces (PP498722-PP498727), and black-and-white ruffed lemur saliva (PP498728-PP498731). While some cressdnaviruses in this study are clustered with publicly available unclassified cressdnaviruses in CRESSV1, CRESSV6, and UC1-7, others represent singletons within the *Cressdnaviricota* phylum.

**Figure 11:**
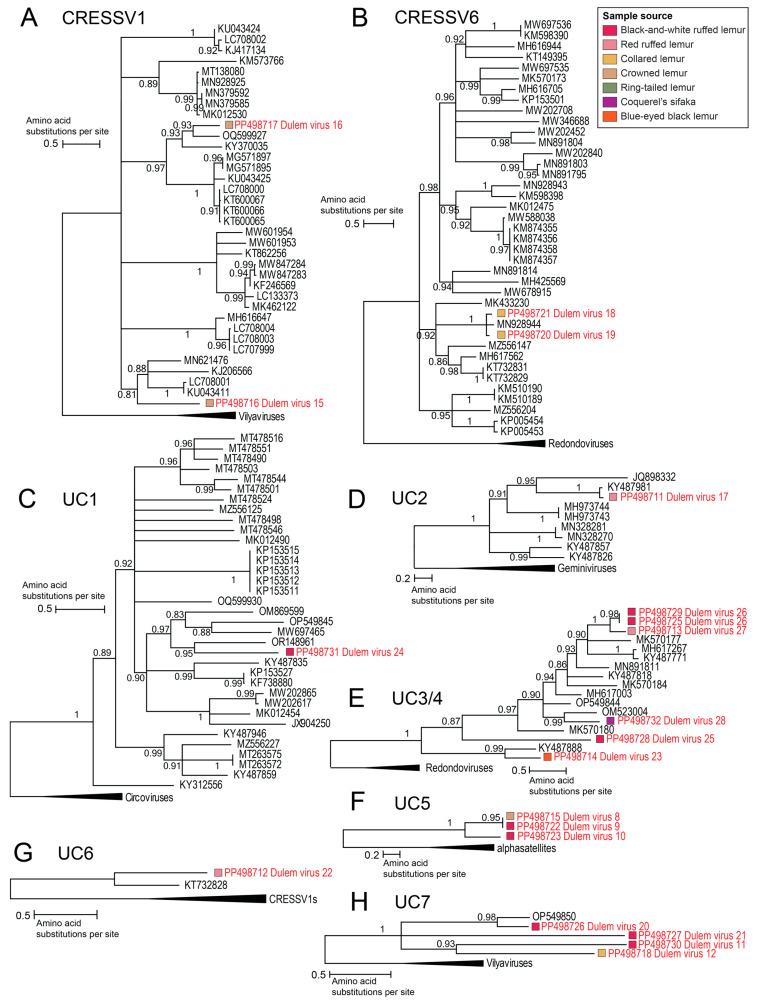
Maximum likelihood phylogenetic trees of the Reps of viruses in the following groups are shown: (**A**) Unclassified CRESSV1 cluster rooted with representative Rep sequences of vilyaviruses. (**B**) Unclassified CRESSV6 cluster rooted with representative Rep sequences of redondoviruses. (**C**) Unclassified cluster UC1 rooted with representative Rep sequences of circoviruses. (**D**) Unclassified cluster UC2 rooted with representative Rep sequences of geminiviruses. (**E**) Unclassified clusters UC3 and UC4 rooted with representative Rep sequences of redondoviruses. (**F**) Unclassified cluster UC5 rooted with representative Rep sequences of alphasatellites. (**G**) Unclassified cluster UC6 rooted with representative Rep sequences from unclassified cluster CRESSV1. (**H**) Unclassified cluster UC7 and 3 neighboring singletons rooted with representative Rep sequences of vilyaviruses. Virus genomes identified in this study are depicted in red font.

Members of the cluster CRESSV1 are predicted to infect *Blastocystis* sp., an environmentally resistant, enteric protozoa parasitizing humans and non-human animals [122]. *Blastocystis* has been detected in studies on parasites in captive lemurs [123]. Within CRESSV1, DlemV16 (PP498717) shares 62% Rep amino acid identity with its closest neighbor banfec virus 3 (OQ599927) from coyote feces [124]. DlemV15 (PP498716) shares its highest Rep identity of 52% with a pecovirus (UC708001) from human stool [122] (Figure 11A). DlemV16 and DlemV15 were identified in one crowned lemur individual’s feces showing the presence of two diverse *Blastocystis*-infecting cressdnaviruses in the lemur’s feces, which suggests a likely *Blastocystis* infection in the lemur. 

For all other unclassified cressdnavirus clusters described here, the host remains unknown. Within CRESSV6, DlemV18 (PP498721) and DlemV19 (PP498720), from a collared lemur fecal sample, share 90% Rep amino acid identity with each other and 88–92% Rep amino acid identity with their closest neighbor, *Cressdnaviricota* sp. isolate fmg067cre2 (MN928944), from a flamingo cloacal swab [125] (Figure 11B). 

We also identified viruses in clusters UC1-7. In UC1, DlemV24 (PP498731) from black-and-white ruffed lemur saliva shares 44% Rep amino acid identity with Cressdnavirus D_HF4_2562 (OR148961) from a passive drool sample taken from DLC staff as part of this larger study [3] (Figure 11C). For UC1, the potential host may be an organism residing in the oral cavity or infecting a food source of people and frugivorous black-and-white ruffed lemurs. 

In UC2, DlemV17 (PP498711) shares 94% Rep amino acid identity with an uncultured virus (KY487981) from wastewater in Florida (USA) [126] (Figure 11D). In UC3, DlemV26 PP498729 and PP498725 share >99% Rep amino acid identity with one another and 75% Rep amino acid identity with DlemV27 (PP498713). DlemV26 and DlemV27 share 50–56% Rep amino acid identity with the closest neighboring cluster of capybara virus 15_cap1_294 (MK570177) [40], *Cressdnaviricota* sp. isolate ctbd281 (MH617267) [127], and uncultured virus CG100 (KY487771) [126] from capybara feces, haddock tissue, and wastewater, respectively (Figure 11E). DlemV28 (PP498732) shares the highest Rep amino acid identity of 52% Rep with chifec virus UA13_133 (OM523004) from Mexican free-tailed bat feces [41] (Figure 11E). The Rep of DlemV25 (PP498728) shares 30% amino acid identity with *Kummerowia striata* CRESS virus strain pt119-gem-1 (MN891811) [128] (Figure 11E). In UC4, the Rep of DlemV23 (PP498714) shares 53% amino acid identity with its closest neighbor uncultured virus CG219 (KY487888) from wastewater [126] (Figure 11E).

In UC5, the Reps of DlemV8 (PP498715), DlemV9 (PP498722), and DlemV10 (PP498723) form a distinct cluster (Figure 11F). The Rep of DlemV8 shares >99% amino acid identity with that of DlemV9 and the Reps of both share 51% amino acid identity with that of DlemV10. In UC6, the Rep of DlemV22 (PP498712) shares 47.1% amino acid identity with that of its closest neighbor Pacific flying fox feces-associated circular DNA virus-11 isolate Tbat_H_102636 (KT732828) [129] (Figure 11G). In UC7, DlemV20’s Rep (PP498726) shares 70% amino acid identity with that of wigfec virus K19_443 (OP549850) from American wigeon feces [130] (Figure 11H). 

DlemV12 (PP498718, 3910 nt in length), DlemV13 (PP498719, 3293 nt), DlemV14 (PP498724, 3100 nt), DlemV21 (PP498727, 2359 nt), DlemV25 (PP498728, 1908 nt), and DlemV11 (PP498730, 4947 nt) represent singletons within the *Cressdnaviricota* phylum that cannot be placed within current family-level clusters. DlemV12 and DlemV13 were identified from collared lemur feces. DlemV14 and DlemV21 were identified from black-and-white ruffed lemur feces and DlemV25 and DlemV11 from black-and-white ruffed lemur saliva. Based on NCBI BLASTp, the Rep of DlemV12 shares 42.2% amino acid identity (query cover 77%) with that of wastewater circular virus FL21 (KX259414) from Florida (USA) [126]. DlemV13’s Rep shares 38.5% amino acid identity (query cover 83%) with that of uncultured virus CG151 (KY487820) from Florida (USA) wastewater [126]. The Rep of DlemV14 shares 41.3% amino acid identity (87% query cover) with that of capybara virus 19_cap1_382 (MK570181) [40]. The Rep of DlemV21 shares 34.6% amino acid identity (78% query cover) with that of *Cressdnaviricota* sp. isolate Miresoil virus 557 (OM154279) from soil in Sweden [131]. The Rep of DlemV25 shares 34.9% amino acid identity (97% query cover) with the Rep of *Cressdnaviricota* sp. Miresoil virus 545 (OM154291) from soil in Sweden [131]. DlemV11’s Rep shares 40.87% amino acid identity (query cover 62%) with that of arizlama virus (MW697506) from lake water (USA). Through time, candidate hosts may be uncovered as more genomes are discovered in different sample types and as unclassified cressdnaviruses begin to form more distinct clusters.

### 3.3. Microviruses in Fecal and Oral Samples

As bacteriophages impact the behavior and evolution of bacteria within the mammalian body, they play a crucial role in the maintenance of mammalian health [132]. In humans, the phageome is impacted by dietary, maternal, and environmental factors, with particular fluctuation seen during phageome establishment in infants [133]. In non-human primates, the phageome has been found to be strongly influenced by both superhost (i.e., sample source species) phylogeny and the captive environment, with captive primates demonstrating a phageome intermediate to conspecific wild populations and human captive facility caretakers [134]. 

Microviruses are ssDNA bacteriophages with small, circular genomes ~4–6 kb in length found ubiquitously across diverse ecosystems [135]. Microvirus genomes generally encode a conserved major capsid protein (MCP), replication initiator protein (Rep), and scaffolding proteins [135,136,137]. For non-human primates, microvirus genomes have been characterized from chimpanzee (*Pan troglodytes* [138]) and macaque (*Macaca mulatta* [138], *Macaca fascicularis* [13]) samples. In one study, microviruses were found to comprise 70% of total viral reads in the feces of cynomolgus macaques [13]. As microviruses had not been previously identified in the lemuriform primates, the hundreds of microvirus genomes characterized in this study serve as the basis of our understanding of circular, ssDNA bacteriophages in lemurs. 

**Figure 12:**
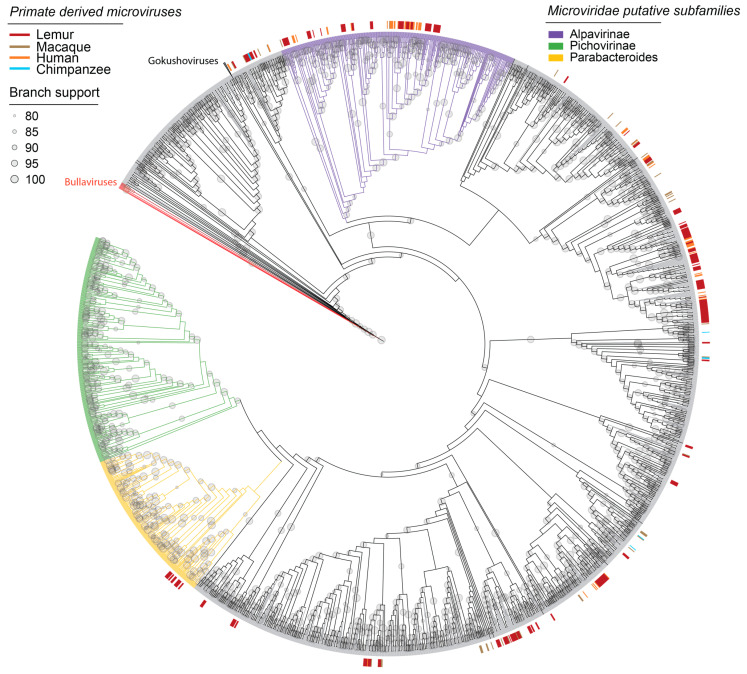
A maximum likelihood phylogenetic tree of major capsid protein sequences of viruses within the *Microviridae* family rooted with representative sequences of bullaviruses. Tree branches are colored by putative subfamilies. Primate-derived microviruses from previous studies (human, chimpanzee, macaque) and from this study (lemurs, in red) are denoted as short lines around the outer edge of the phylogeny.

In this study, 537 complete microvirus genomes were characterized from collared lemur (*n* = 60 microvirus genomes), crowned lemur (*n* = 48), blue-eyed black lemur (*n* = 9), ring-tailed lemur (*n* = 65), Coquerel’s sifaka (*n* = 78), and black-and-white ruffed lemur (*n* = 236) feces. Complete microvirus genomes were also identified in the saliva of black-and-white ruffed lemurs (*n* = 20 microvirus genomes) and red ruffed lemurs (*n* = 21). Microvirus genomes ranged in length from 4057 to 6727 nt and in GC content from 28.6 to 58.6%. The majority of microvirus genomes characterized in this study fall within the Alpavirinae and Parabacteroides putative subfamilies or unclassified clades within *Microviridae* (Figure 12). Alpavirinae and unclassified clades contain most primate-derived microviruses that have been characterized thus far. Lemur-derived microviruses were found to cluster with other primate-derived microviruses from human, macaque, and chimpanzee samples (Figure 12). 

**Figure 13:**
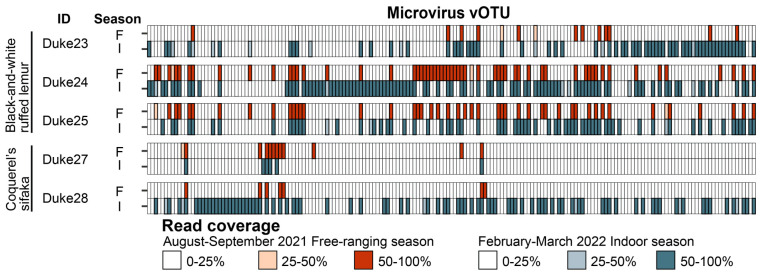
Microvirus composition differs across individuals and sample species. For three black-and-white ruffed lemurs (Duke23, Duke24, and Duke25) and two Coquerel’s sifakas (Duke27, Duke28), we collected fecal samples in both the free-ranging season (F) during the warmer months of the year and the indoor season (I) during the colder months of the year. For each individual, microvirus presence (high confidence is 50–100% read coverage) is shown for each sample taken during the free-ranging season (dark red) and the indoor season (dark gray-blue).

For three black-and-white ruffed lemurs and two Coquerel’s sifakas, we collected fecal samples in both the free-ranging season during the warmer months of the year and the indoor season during the colder months of the year when the lemurs cannot free-range. While microvirus composition was not statistically impacted by season, black-and-white ruffed lemur and Coquerel’s sifaka fecal microvirus vOTU richness, Shannon’s diversity, and Simpson’s diversity metrics did differ statistically across species (Wilcoxon signed-rank test, *p* = 0.038) (Figure 13). As we only had one fecal sample each for collared lemur, crowned lemur, ring-tailed lemur, and blue-eyed black lemur, this limited our ability to look at differences across these species. 

**Figure 14:**
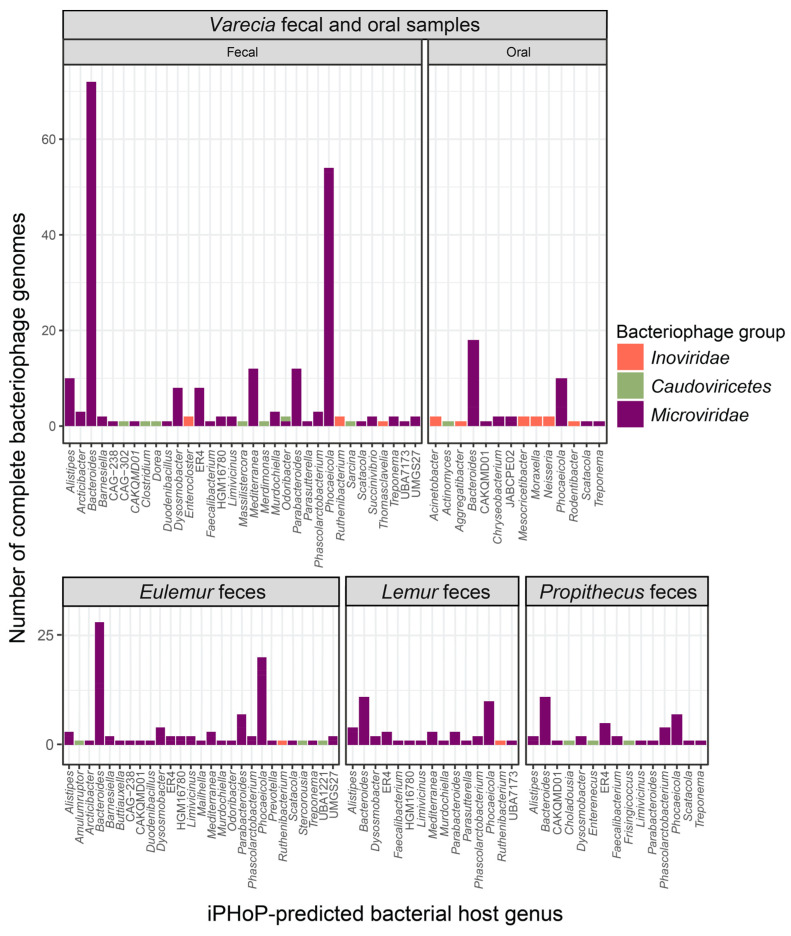
The predicted bacterial host genus, determined from iPHoP [50], of complete bacteriophage genomes from the *Inoviridae* and *Microviridae* families and *Caudoviricetes* order identified in this study from fecal and oral samples of diverse lemur genera (*Varecia*, *Eulemur*, *Lemur*, *Propithecus*). This figure only includes phage genomes for which iPHoP was able to determine a predicted host. Some phage genomes were predicted, with high confidence (>90% iPHoP confidence score), to infect bacteria across multiple genera.

Based on iPHoP [50] analysis of predicted hosts with >90% confidence scores, microviruses in lemur fecal samples were predicted to infect bacteria within the genera *Alistipes*, *Arcticibacter*, *Bacteroides*, *Barnesiella*, *Buttiauxella*, *Duodenibacillus*, *Dysosmobacter*, *Faecalibacterium*, *Limivicinus*, *Mailhella*, *Mediterranea*, *Murdochiella*, *Odoribacter*, *Parabacteroides*, *Parasutterella*, *Phascolarctobacterium*, *Phocaeicola*, *Prevotella*, *Scatacola*, *Succinivibrio*, and *Treponema* (Figure 14; Table S3). Additionally, microviruses in lemur oral samples were predicted to infect bacteria within the genera *Bacteroides*, *Chryseobacterium*, *Phocaeicola*, *Scatacola*, and *Treponema* (Figure 14; Table S3). 

Our study emphasizes the significant diversity of microviruses within even a single primate sample (e.g., 65 complete, distinct microvirus genomes from one ring-tailed lemur fecal sample). Additionally, many of the same microvirus species were found across multiple lemur species (e.g., DlemV29 was present across blue-eyed black lemur, collared lemur, crowned lemur, ring-tailed lemur, black-and-white ruffed lemur, and Coquerel’s sifaka fecal and oral samples; Figure 3), although, microvirus vOTU diversity metrics overall differed between Coquerel’s sifaka and black-and-white ruffed lemurs, species for which we had multiple samples. 

### 3.4. Inoviruses in Fecal and Oral Samples

Viruses in the *Inoviridae* family have ssDNA genomes with unique filamentous capsid morphology, and they are known to impact bacterial toxicity and growth. For example, inoviruses have been found to inhibit biofilms of *Aspergillus fumigatus* [139] and *Candida albicans* [140]. Further, inoviruses can establish chronic infections in bacterial hosts, as host cell lysis is not required for infectious virion release [141,142]. Inoviruses have circular genomes ~5.5–10.6 kb in length encoding 7–15 proteins [141]. Diverse inoviruses have been identified in primate (e.g., human [3], rhesus macaque [143]) oral and fecal samples primarily through metagenomics. However, prior to this study, there were no sequences of inoviruses identified from lemur samples. 

**Figure 15:**
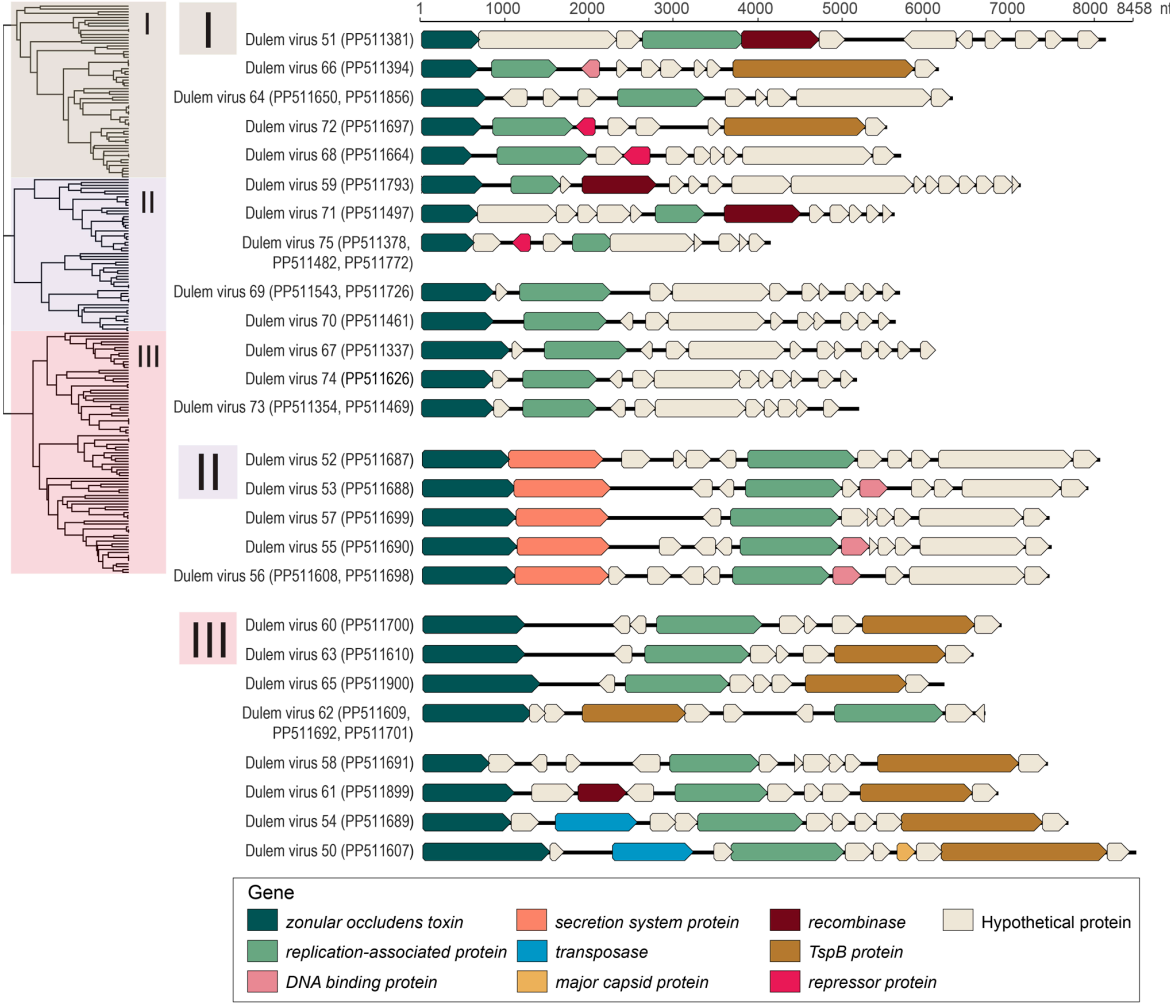
Genome organizations for inoviruses identified in this study in the order in which they appear in the ViPTree phylogeny (Figure 16). Accession numbers of genomes displayed in the same row share >98% nt similarity.

Thirty-four complete inovirus genomes were identified from lemur fecal and oral samples. Complete inovirus genomes were identified from crowned lemur (*n* = 3 inovirus genomes), collared lemur (*n* = 2), ring-tailed lemur (*n* = 2), Coquerel’s sifaka (*n* = 1), and black-and-white ruffed lemur (*n* = 9) feces. Additionally, complete inovirus genomes were identified in the saliva of red ruffed lemurs (*n* = 2 inovirus genomes) and black-and-white ruffed lemurs (*n* = 15). Inovirus genomes recovered in this study ranged in length from 4134 to 8458 nt and in GC content from 23.8 to 51.7%. All inovirus genomes contained at minimum an identified zonula occludens toxin protein and replication protein (Figure 15). 

**Figure 16:**
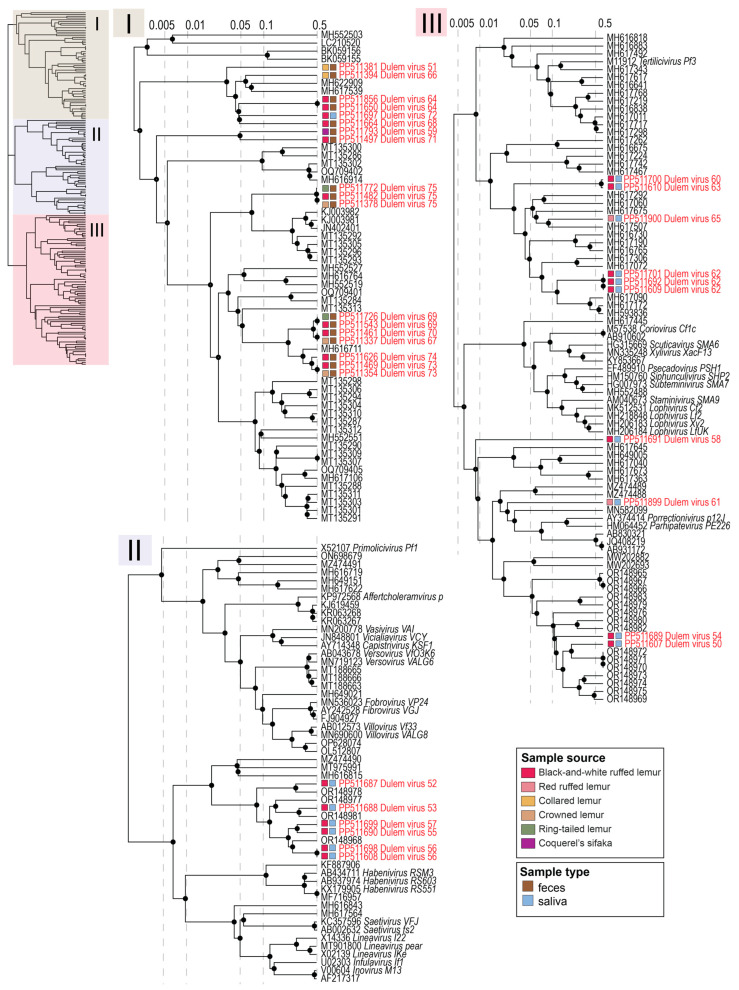
ViPTree proteomic phylogeny of viruses in the *Inoviridae* family. The phylogeny is divided into three clades, I (tan), II (purple), and III (light red), for visualization. Viruses identified in this study from lemur fecal and oral samples are shown in red font. The sample source species and sample type are shown in boxes next to each virus identified in this study.

As seen in Figure 16, multiple clusters of inovirus genomes shared >98% similarity, suggesting they are the same inovirus species. The same inovirus species were frequently found in the samples of diverse lemurs, including lemurs across multiple genera (Figure 3). For example, DlemV75 (PP511378) was identified in crowned lemur, collared lemur, ring-tailed lemur, and black-and-white ruffed lemur feces, and DlemV73 (PP511354) was present in crowned lemur, collared lemur, black-and-white ruffed lemur, and Coquerel’s sifaka feces. Inovirus genomes identified from fecal samples all clustered in clade I of the inovirus VipTree phylogeny (Figure 16). 

Inovirus genomes identified in lemur fecal samples were predicted to infect bacteria in the genera *Ruthenibacterium*, *Enterocloster*, and *Thomasclavia* through iPHoP [50] analysis (Table S3). Inovirus genomes characterized from oral samples clustered in clades II and III of the inovirus VipTree phylogeny with the exception of DlemV72 (PP511697), which clustered with clade I (Figure 16). As clade I contained the inoviruses found in lemur fecal samples, it is possible that individual Duke_25 may have had fecal material in its mouth from coprophagic behavior. This is additionally supported by a smacovirus present in the same sample. Inovirus genomes identified in lemur oral samples were predicted to infect bacteria in the genera *Mesocricetibacter*, *Acinetobacter*, *Moraxella*, *Neisseria*, *Rodentibacter*, and *Aggregatibacter* based on iPHoP [50] analysis (Table S2). Lemur-associated inovirus genomes within each clade, especially clade II, demonstrate similar genomic structure; however, inovirus genomes within clade I demonstrate varied genome size (Figure 15). Intergenomic similarities between the inoviruses in clades I, II, and III are shown in Figures S1–S4. The distinct phylogenetic separation between fecal and oral inoviruses reflects differences in bacterial host communities in the gastrointestinal tract versus oral cavity. 

### 3.5. Caudoviruses in Fecal and Oral Samples

**Figure 17:**
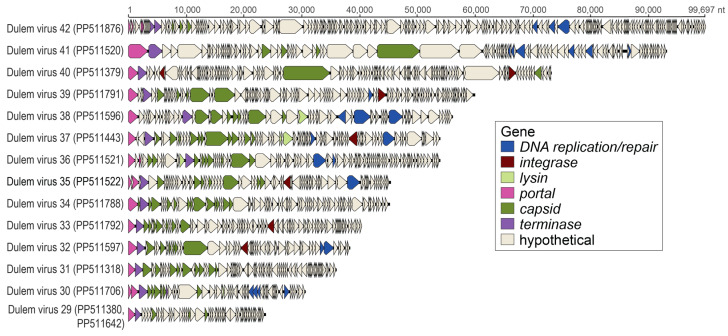
Linearized genome organizations of caudoviruses identified in this study.

**Figure 18:**
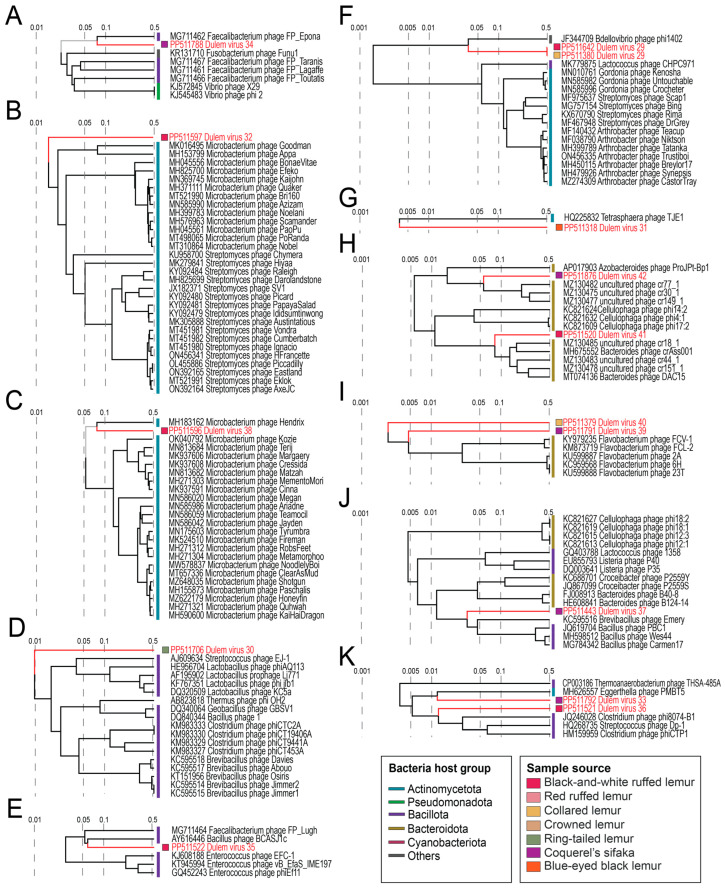
A proteomic tree, generated with ViPTree, and annotations of caudovirus genomes. Subsets of the phylogeny, shown in (**A**–**K**), depict each clade containing genomes identified in this study.

Tailed, dsDNA bacteriophages in the class *Caudoviricetes* have large genomes varying widely in structure and size. For all 15 *Caudoviricetes* bacteriophages identified in this study, we were able to identify at minimum the terminase, portal protein, and structural proteins (Figure 17). Large dsDNA bacteriophage genomes were identified from the feces of a collared lemur (*n* = 2 phage genomes), a blue-eyed black lemur (*n* = 1), a ring-tailed lemur (*n* = 1), Coquerel’s sifakas (*n* = 5), and black-and-white ruffed lemurs (*n* = 4) and from the saliva of black-and-white ruffed lemurs (*n* = 2). Large bacteriophage genomes ranged from 23,551 nt to 99,697 nt in length and with a 31.5 to 67.8% range in GC content (Figure 17). DlemV34 (PP511788), from Coquerel’s sifaka feces, is most closely related to Faecalibacterium phage FP_Epona (MG711462) (Figure 18A). DlemV32 (PP511597), from black-and-white ruffed lemur saliva, clusters with Microbacterium phages (e.g., MK016495, MT310864) and Streptomyces phages (e.g., KU958700, MK305888) (Figure 18B). DlemV38 (PP511596), from black-and-white ruffed lemur saliva, clusters with a Microbacterium phage Hendrix (MH183162) (Figure 18C) and is predicted to infect *Actinomyces* sp. (Table S5). DlemV38 was present in the saliva of four black-and-white ruffed lemur individuals’ samples and one red ruffed lemur sample, suggesting that *Actinomyces* sp. may be an important player in the lemur oral microbiome (Figure 3). DlemV30 (PP511706), from ring-tailed lemur feces and predicted to infect *Sarcina* sp. (Table S5), clusters distantly with Streptococcus phage EJ-1 (AJ609634), Lactobacillus phages (e.g., HE956704), Thermus phage phi OH2 (AB823818), Geobacillus phage GBSV1 (DQ340064), Bacillus phage1 (DQ840344), Clostridium phages (e.g., KM983333), and Brevibacillus phages (e.g., KC595515) (Figure 18D). DlemV35 (PP511522), from black-and-white ruffed lemur feces, clusters with Faecalibacterium phage FP_Lugh (MG711464) and Bacillus phage BCASJ1c (AY616446) (Figure 18E). 

DlemV29 genomes (PP511642; PP511380) share >98% nt similarity with one another and are related to a Bdellovibrio phage phi1402 (JF344709) (Figure 18F). DlemV31 (PP511318), from blue-eyed black lemur feces, is distantly related to a Tetrasphaera phage TJE1 (HQ225832) (Figure 18G) and predicted to infect bacteria in the genus *Stercorousia* (Table S5). DlemV42 (PP511876), from Coquerel’s sifaka feces, and DlemV41 (PP511520), from black-and-white ruffed lemur feces and predicted to infect *Odoribacter* sp. (Table S5), fall within the same clade (Figure 18H). DlemV42 is more closely related to Azobacteroides phage ProJPt-Bp1 (AP017903) and uncultured phages (e.g., MZ130482), while DlemV41 is more closely related to Bacteroides phages (MH675552, MT074136) (Figure 18H). DlemV40 (PP511379), from collared lemur feces and predicted to infect *Amulumruptor* sp. (Table S5), and DlemV39 (PP511791), from Coquerel’s sifaka feces and predicted to infect *Frisingicoccus* sp. (Table S5), cluster with Flavobacterium phages (e.g., KY979235, KC959568) (Figure 18I). DlemV37 (PP511443), present in three Coquerel’s sifaka individuals’ feces from the free-ranging season (Figure 3) and predicted to infect *Choladousia* sp. (Table S5), clusters with Brevibacillus phage Emery (KC595516) and Bacillus phages (JQ619704, MH598512, MG784342) (Figure 18J). DlemV36 (PP511521) from black-and-white ruffed lemur feces was predicted to infect *Massilistercora* sp., *Dorea* sp., *Merdimonas* sp., and *Clostridium* sp. (Table S5), and DlemV33 (PP511792) from Coquerel’s sifaka feces was predicted to infect *Enterenecus* sp. (Table S5). DlemV36 and DlemV33 cluster with Clostridium phage phi8074-B1 (JQ246028), Streptococcus phage Dp-1 (HQ268735), Eggerthella phage PMBT5 (MH626557), and Thermoanaerobacterium phage THSA-485A (CP003186) (Figure 18K). 

Although the caudoviruses in this study phylogenetically fall within known clusters, all caudoviruses identified from lemur fecal and oral samples are highly divergent from known large phages. Based on VIRIDIC analyses, the lemur-associated caudoviruses share <10% intergenomic similarity with known caudoviruses within their respective clusters (Figures S5–S15). Numerous caudoviruses identified in this study were present across multiple species (e.g., DlemV29 and DlemV30 were present in collared lemur, crowned lemur, and black-and-white ruffed lemur feces; DlemV36 was present across black-and-white ruffed lemur and ring-tailed lemur feces; DlemV39 was present in black-and-white ruffed lemur and Coquerel’s sifaka feces; Figure 3). DlemV33 and DlemV39 were present across Lemuridae and Indriidae samples as they were characterized from Coquerel’s sifaka and black-and-white ruffed lemur feces. The presence of the same phage species across lemur species and diverse lemur families suggests that for many captive lemur phages (*Caudoviricetes*, *Microviridae*, and *Inoviridae*), sample source species phylogeny may be less important than the impacts of the captive environment. However, the presence of some phages appears to differ by sample species and season (i.e., free-ranging outdoor or indoor season in captivity). DlemV34 was present only in the free-ranging season fecal samples for Coquerel’s sifaka individuals Duke_22, Duke_27, Duke_28, and Duke_30. Similarly, DlemV37 was only in the free-ranging fecal samples of Duke_22, Duke_28, and Duke_30. DlemV34 and DlemV37 may be phages associated with the gut bacteria required for sifaka to consume different plant materials during the free-ranging season. Coquerel’s sifaka at the DLC eat a higher quantity of leaves from a more diverse array of trees during the free-ranging season [144]. 

## 4. Conclusions

As lemurs are one of the largest primate lineages and at unusually high risk of extinction, extensive research goes into maintaining captive lemur populations globally. However, there is a scarcity of research on the virome of captive or wild lemur populations despite their phylogenetic and conservation importance. To gain some insight into the DNA viromes of lemurs, this study investigated captive lemur fecal, blood, and oral viral communities across collared lemur, crowned lemur, blue-eyed black lemur, ring-tailed lemur, Coquerel’s sifaka, black-and-white ruffed lemur, and red ruffed lemur samples. In this study, we identified diverse anelloviruses, cressdnaviruses, microviruses, inoviruses, and caudoviruses, forming foundational knowledge of lemur-associated viruses across different sample types (Figure 1). While anelloviruses in this study are species-specific vertebrate-infecting viruses, the same cressdnaviruses (i.e., smacoviruses, vilyaviruses) and bacteriophages (i.e., caudoviruses, microviruses, inoviruses) were found to be present across multiple lemur species spanning lemur families (Lemuridae and Indriidae). The lemur virome is likely impacted by various behavioral and environmental influences, such as aggressive (e.g., conflict with conspecifics), social (e.g., grooming with tooth comb, scent-marking, group size), and dietary (e.g., geophagy, coprophagy) behaviors, as well as lemur physiology and anatomy (e.g., gastrointestinal tract length in folivores versus frugivores). Further, the DNA viromes of captive lemurs may be affected by close, extended contact with conspecifics, heterospecifics, and caretakers along with animal husbandry decisions, including diet and access to outdoor forested areas. Overall, this study is a first look into the vast diversity of vertebrate- and nonvertebrate-infecting viruses in lemuriform primates. Future research will examine and compare viral communities of wild lemur populations. 

## Data Availability

The sequences described in this study are deposited in GenBank under accession numbers PP498706-PP498756 and PP511318-PP511903. The raw reads are deposited in SRA under BioProject number PRJNA956591; BioSample numbers: SAMN40214160-SAMN40214201; SRA accession numbers: SRS20648727- SRS20648768.

## Supplementary Materials

The following supporting information can be downloaded at: https://www.mdpi.com/article/10.3390/v16071099/s1, Table S1. Summary table of virus genomes identified per sample; Table S2: Motifs from cressdnavirus Reps identified in study; Table S3: Microvirus iPHoP predictions; Table S4: Inovirus iPHoP predictions; Figure S1: VIRIDIC heatmap from a subset of the Inoviridae phylogeny of clade ‘A’ including inoviruses identified in this study; Figure S2: VIRIDIC heatmap from a subset of the *Inoviridae* phylogeny of clade ‘B’ including inoviruses identified in this study; Figure S3: VIRIDIC heatmap from a subset of the *Inoviridae* phylogeny of clade ‘C’ including inoviruses identified in this study; Figure S4: VIRIDIC heatmap from a second subset of the *Inoviridae* phylogeny of clade ‘C’ including inoviruses identified in this study; Table S5: Caudovirus iPHoP predictions; Figure S5: VIRIDIC heatmap from a subset of the caudovirus phylogeny from Figure 18A; Figure S6: VIRIDIC heatmap from a subset of the caudovirus phylogeny from Figure 18B; Figure S7: VIRIDIC heatmap from a subset of the caudovirus phylogeny from Figure 18C; Figure S8: VIRIDIC heatmap from a subset of the caudovirus phylogeny from Figure 18D; Figure S9: VIRIDIC heatmap from a subset of the caudovirus phylogeny from Figure 18E; Figure S10: VIRIDIC heatmap from a subset of the caudovirus phylogeny from Figure 18F; Figure S11: VIRIDIC heatmap from a subset of the caudovirus phylogeny from Figure 18G; Figure S12: VIRIDIC heatmap from a subset of the caudovirus phylogeny from Figure 18H; Figure S13: VIRIDIC heatmap from a subset of the caudovirus phylogeny from Figure 18I; Figure S14: VIRIDIC heatmap from a subset of the caudovirus phylogeny from Figure 18J; Figure S15: VIRIDIC heatmap from a subset of the caudovirus phylogeny from Figure 18K.
