# Supplementary material for: Diverse Circular DNA Viral Communities in Blood, Oral, and Fecal Samples of Captive Lemurs
Source: Viruses. 2024 Jul 8;16(7):1099. doi: 10.3390/v16071099 (PMC11281440; doi:10.3390/v16071099)
Supplement: Supplementary file 1 [file viruses-16-01099-s001.zip › viruses-3078293-supplementary-conversion.pdf]

Supplementary Material for:

Diverse circular DNA viral communities in blood, oral, and fecal samples of captive lemurs

Elise N Paietta, Simona Kraberger, Michael Lund, Karla L Vargas, Joy M Custer, Erin E. Ehmke, Anne D. Yoder, & Arvind Varsani

Table of Contents

I. **Table S1.** Summary table of virus genomes identified per sample
II. **Table S2.** Motifs from cressdnavirus Reps identified in this study
III. **Table S3.** Microvirus iPHoP predictions
IV. **Table S4.** Inovirus iPHoP predictions
V. **Figure S1.** VIRIDIC heatmap from a subset of the *Inoviridae* phylogeny of clade 'A' including inoviruses identified in this study.
VI. **Figure S2.** VIRIDIC heatmap from a subset of the *Inoviridae* phylogeny of clade 'B' including inoviruses identified in this study.
VII. **Figure S3.** VIRIDIC heatmap from a subset of the *Inoviridae* phylogeny of clade 'C' including inoviruses identified in this study.
VIII. **Figure S4.** VIRIDIC heatmap from a second subset of the *Inoviridae* phylogeny of clade 'C' including inoviruses identified in this study.
IX. **Table S5.** Caudovirus iPHoP predictions
X. **Figure S5.** VIRIDIC heatmap from a subset of the caudovirus phylogeny from Figure 18A.
XI. **Figure S6.** VIRIDIC heatmap from a subset of the caudovirus phylogeny from Figure 18B.
XII. **Figure S7.** VIRIDIC heatmap from a subset of the caudovirus phylogeny from Figure 18C.
XIII. **Figure S8.** VIRIDIC heatmap from a subset of the caudovirus phylogeny from Figure 18D.
XIV. **Figure S9.** VIRIDIC heatmap from a subset of the caudovirus phylogeny from Figure 18E.
XV. **Figure S10.** VIRIDIC heatmap from a subset of the caudovirus phylogeny from Figure 18F.
XVI. **Figure S11.** VIRIDIC heatmap from a subset of the caudovirus phylogeny from Figure 18G.
XVII. **Figure S12.** VIRIDIC heatmap from a subset of the caudovirus phylogeny from Figure 18H.
XVIII. **Figure S13.** VIRIDIC heatmap from a subset of the caudovirus phylogeny from Figure 18I.

XIX. **Figure S14.** VIRIDIC heatmap from a subset of the caudovirus phylogeny from Figure 18J.

XX. **Figure S15.** VIRIDIC heatmap from a subset of the caudovirus phylogeny from Figure 18K.

Table S1. Summary table of complete virus genomes identified per sample

Sample ID	Species	Sample Type	Number of Complete Virus Genomes Identified in Each Sample								
			Anelloviridae	*Smacoviridae*	*Genomoviridae*	*Geminiviridae*	*Vilyaviridae*	Unclassified cressdnavirus	*Microviridae*	*Inoviridae*	*Caudoviricetes*
Duke_17	*Eulemur flavifrons*	fecal	1	1	0	0	0	1	9	0	1
Duke_18	*Eulemur coronatus*	fecal	0	2	0	0	0	3	48	3	0
Duke_21	*Eulemur collaris*	fecal	0	1	0	0	0	4	60	2	2
Duke_22FF	*Propithecus coquereli*	fecal	0	0	0	0	0	0	2	0	1
Duke_23BS	*Varecia variegata*	blood	2	0	0	0	0	0	0	0	0
Duke_23FF	*Varecia variegata*	fecal	0	0	0	0	0	0	2	0	0
Duke_23FS	*Varecia variegata*	fecal	0	2	0	0	0	2	32	3	0
Duke_23SF	*Varecia variegata*	saliva	1	0	0	0	0	0	0	0	0
Duke_24FF	*Varecia variegata*	fecal	0	2	0	0	0	2	36	1	0
Duke_24FS	*Varecia variegata*	fecal	0	0	1	0	1	0	72	1	3
Duke_24SF	*Varecia variegata*	saliva	0	0	0	0	0	0	9	0	2
Duke_24SS	*Varecia variegata*	saliva	0	0	0	0	0	0	3	4	0
Duke_25FF	*Varecia variegata*	fecal	0	1	0	0	0	2	27	1	0
Duke_25FS	*Varecia variegata*	fecal	0	1	0	0	0	0	42	2	1
Duke_25SF	*Varecia variegata*	saliva	0	1	0	0	0	2	4	7	0
Duke_25SS	*Varecia variegata*	saliva	0	0	0	0	0	2	4	4	0
Duke_26	*Lemur catta*	fecal	0	1	0	0	1	0	65	2	1
Duke_27FF	*Propithecus coquereli*	fecal	0	0	0	0	0	1	9	0	0
Duke_27FS	*Propithecus coquereli*	fecal	0	0	1	0	0	0	5	0	0
Duke_28FF	*Propithecus coquereli*	fecal	0	0	0	0	0	0	2	0	1
Duke_28FS	*Propithecus coquereli*	fecal	0	1	0	0	0	0	56	1	2
Duke_29	*Varecia variegata*	fecal	0	1	0	0	0	0	25	1	0
Duke_30FF	*Propithecus coquereli*	fecal	0	0	0	0	0	0	4	0	1
Duke_43	*Varecia rubra*	saliva	0	1	2	1	0	3	18	0	0
Duke_44	*Varecia rubra*	saliva	0	0	2	0	0	0	3	2	0
Duke_46	*Varecia rubra*	saliva	0	0	1	0	0	0	0	0	0

Table S2. Rep motifs of cressdnavirus identified in this study.

Family/cluster	Genus	Accession	Motif I	Motif II	GRS	Motif III	Walker A	Walker B	Motif C	Arg finger
Smacoviridae	*Porprismacovirus*	PP498740	MMTAPR	KHWQI		PKWDYEAK	DEGGNVGKSWFTG	FIDIP	VMTN	LSYDRW
		PP498742	MMTAPR	KHWQI		PKWDYEAK	DEGGNVGKSWFTG	FIDIP	VMTN	LSYDRW
		PP498746	MMTAPR	KHWQI		PKWDYEAK	DEGGNVGKSWFTG	FIDIP	VMTN	LSYDRW
		PP498753	MLTIPR	AHWQI		DVWDYERK	DPRGNRGKSWLCG	VIDIP	VLAN	LSVDRW
		PP498744	MLTIPR	AHWQI		DVWDYERK	DPRGNRGKSWLCG	VIDIP	IVTL	
		PP498749	MLTIPR	AHWQI		DVWDYERK	DPRGNRGKSWLCG	VIDIP	IVTL	
		PP498741	MLTIPR	AHWQI		DVWDYERK	DPRGNRGKSWLCG	VIDIP	FVTL	
		PP498751	MLTIPR	AHWQI		DVWDYERK	DPRGNRGKSWLCG	VIDIP	IVTL	
		PP498747	MLTIPR	AHWQI		DVWDYERK	DPRGNRGKSWLCG	VIDIP	IVTL	
		PP498754	FIAIMR	EHWQI		NGWEYEGK	PKGNSGKSWLVG	VIDIP	VLTN	LSADRW
		PP498750	FIAIMR	EHWQI		NGWEYEGK	PKGNSGKSWLVG	VIDIP	VLTN	LSADRW
		PP498745	FIAIMR	EHWQI		NGWEYEGK	PKGNSGKSWLVG	VIDIP	VLTN	LSADRW
		PP498752	MVTMPR	EHWQI		NGWEYEGK	DPKGNSGKSWLVG	VIDIP	VLTN	LSADRW
		PP498748	MVTMPR	EHWQI		NGWEYEGK	DPKGNSGKSWLVG	VIDIP	VLTN	LSADRW
		PP498743	MVTMPR	EHWQI		NGWEYEGK	DPKGNSGKSWLVG	VIDIP	VLTN	LSADRW
Genomoviridae	*Gemycircularvirus*	PP498733	LVTYAQ	FHLHC	DIFDVDGHHPNIEPSR	KGYDYAIK	GESRTGKTLWAR	IFDDI	WISN	WLEANA
	Gemykibivirus	PP498734	LLTYAQ	VHLHA	RVFDVAGRHPNVVAGY	KGWAYATK	GDTRLGKTLWAR	VFDDM	YCCN	WLVGNC
	Gemykrogvirus	PP498739	FLTYSQ	SHLHC	SLFDYRGAHPNIKSIR	KPWNYAGK	GPSRTGKTVWAR	IFDDI	MCMN	WLLLNC

		PP498737	ILTFPQ	VHFHV	DAFNYFGAHGNIKSVR	KVYDYVTK	GPSRTGKTLYAR	VFDDI	MLMN	WLKLNC
		PP498735	ILTFPQ	VHFHV	DAFDYFGAHGNIKSVR	KVYDYVTK	GPSRTGKTLYAR	VFDDI	MLMN	WLKLNC
		PP498736	ILTFPQ	IHFHV	DAFDYFGSHGNIKSVR	KVYDYVGK	GPTRTGKTLFAR	VFDDI	MLMN	WLLGNC
		PP498738	ILTFPQ	IHFHV	DAFDYFGSHGNIKSVR	KVYDYVGK	GPTRTGKTLFAR	VFDDI	MLMN	WLLGNC
Geminiviridae	*Curtovirus*	PP498706	FLTYPR	LHLHA	RHFDITHPSSSPTFHPNFQGAKS	DVKQYIEK	GDSRTGKTMWAR	VIDDV	ILCN	LRSWTL
Vilyaviridae	*Aranruthvirus*	PP498756	CGTLN	WHFQF		QNKAYCMK	GICSTQGKQTGL	ILDDF	ITSN	TVMRRI
		PP498755	CGTLN	WHFQF		QNKAYCMK	GICSTQGKQTGL	ILDDF	ITSN	TVMRRI
CRESSV1		PP498717	CITVFK	EHWHI		DAIDYCKK	GPAGSGKSHHCY	WFDEF	ISTV	QLFRRL
		PP498716	VTTYNI	LHYHM		QARDYVTK	GKSGTGKSHACW	WFDEF	ISTT	QLWRRL
CRESSV6		PP498721	LLTYPR	YHLHA		KCVNYCRK	GPSGAGKTTWAL	VFDEI	TCTD	QIKRRV
		PP498720	LLTYPR	YHLHA		ACVNYCRK	GPSGAGKTTWAL	VFDEI	TCTD	QIKRRV
DC1		PP498731	AYTCNN	AHIQA		AYMAYVSK	GTTGTGKSEWGF	IIDDF	ITCE	QVMRRI
DC2		PP498711	FLTYAQ	IHFHC		NVIKYCTK	GPSRFGKTALAR	VLDDI	NA	RNWLRV
DC3		PP498729	FLTYPQ	HHLHM		NVIKYCTK	SRQPNKGKTYHFA	VLDEY	VLSN	LLYARF
		PP498725	FLTYPQ	HHLHM		NVIKYCTK	SRQPNKGKTYHFA	VLDEY	VLSN	LLYARF
		PP498713	FLTYPQ	FHLHC		NVIKYCTK	RVPNVGKTYHFA	ILDEY	ILSN	LLYARF
		PP498732	FLTWPQ	HHLHA		NVIKYCTK	GPNAGKTTFAES	IIDEF	LLGN	LLYARY
		PP498714	FLTWPKNN	PHLHA		DVHDYVKK	GSPNTGKTSFIN	YVDEF	ILSN	SLKTRL
DC4		PP498715	SFTINI	THIQG		QNYAYCTK	GSAGSGKSMLAD	VLDDL	VTTN	PIRRRF
		PP498722	SFTINI	THIQG		QNYAYCTK	GSAGSGKSMLAD	VLDDL	VTTN	PIRRRF
		PP498723	VFTINN	PHIQG		ANYRYCTK	GSAGSGKSVLPT	VIDDL	ITTN	PIRRRF

DC5	PP498712	CFTLNN	RHLQG	QARDYCRK	GPPGCGKTRRIR	VFDDM	LSSC	EIARRI
DC6	PP498726	LFTLWLHN	RHFQC	QNVNYCSK	GEPGTGKTRFAY	LFDDY	ITSN	ALYRRL
DC7	PP498728	FLTYPK	LHLHA	RTARYCRK	SAPNWGKTTWVE	IYDDY	VLSN	RFLVRF
Singletons	PP498718	VFTLHI	RHLQG	RAKDYAGK	GPTGTGKSRTVD	LFDEC	LCSN	ALKRRV
	PP498719	VFTINN	VHWQG	QARDYFAN	GPPGIGKSSGVI	RMDDF	ITSN	AINRRC
	PP498724	VFTINN	VHWQG	QARDYFAN	GPPGIGKSSGVI	RMDDF	ITSN	AFMRRL
	PP498726	LFTLWLHN	RHFQC	QNVNYCSK	GEPGTGKTRFAY	LFDDY	ITSN	ALYRRL
	PP498727	IYTLWP	PHVQL	RMRYYCSK	GASGSGKTHWVK	LLDDF	ITTN	ALQRRI
	PP498730	MFTVNQ	LHIQG	KAVEYCGK	GSTGTGKTLRAT	VMDDV	ITTN	AVERRI

Table S3. Microvirus iPHoP predictions

Accession No.	Bacterial Host Genus	Confidence (%)	Sample species	Sample type
PP511884	*Phocaeicola*	99.3	*Varecia rubra*	oral
PP511884	*Bacteroides*	98.8	*Varecia rubra*	oral
PP511885	*Bacteroides*	99.6	*Varecia rubra*	oral
PP511885	*Phocaeicola*	99.6	*Varecia rubra*	oral
PP511886	*Bacteroides*	99.7	*Varecia rubra*	oral
PP511886	*Phocaeicola*	99.6	*Varecia rubra*	oral
PP511887	*Bacteroides*	99.6	*Varecia rubra*	oral
PP511887	*Phocaeicola*	99.3	*Varecia rubra*	oral
PP511901	JABCPE02	98.4	*Varecia rubra*	oral
PP511901	*Chryseobacterium*	97	*Varecia rubra*	oral
PP511319	*Prevotella*	93.2	*Eulemur flavifrons*	fecal
PP511320	*Phocaeicola*	97.6	*Eulemur flavifrons*	fecal
PP511320	*Bacteroides*	97	*Eulemur flavifrons*	fecal
PP511321	*Buttiauxella*	91.8	*Eulemur flavifrons*	fecal
PP511322	*Bacteroides*	99.7	*Eulemur flavifrons*	fecal
PP511322	*Phocaeicola*	99.6	*Eulemur flavifrons*	fecal
PP511323	*Bacteroides*	99.6	*Eulemur flavifrons*	fecal
PP511323	*Phocaeicola*	99.6	*Eulemur flavifrons*	fecal
PP511324	*Limivicinus*	94	*Eulemur flavifrons*	fecal
PP511328	*Phocaeicola*	97.8	*Eulemur coronatus*	fecal
PP511328	*Bacteroides*	97.2	*Eulemur coronatus*	fecal
PP511329	*Bacteroides*	98.4	*Eulemur coronatus*	fecal
PP511330	*Bacteroides*	99.7	*Eulemur coronatus*	fecal
PP511330	*Phocaeicola*	99.7	*Eulemur coronatus*	fecal
PP511330	*Murdochiella*	91	*Eulemur coronatus*	fecal
PP511332	*Bacteroides*	99.6	*Eulemur coronatus*	fecal
PP511332	*Phocaeicola*	99.3	*Eulemur coronatus*	fecal
PP511333	*Bacteroides*	99.7	*Eulemur coronatus*	fecal
PP511333	*Phocaeicola*	99.7	*Eulemur coronatus*	fecal
PP511335	*Bacteroides*	99.1	*Eulemur coronatus*	fecal
PP511335	*Phocaeicola*	99.1	*Eulemur coronatus*	fecal
PP511335	*Bacteroides_I*	94.9	*Eulemur coronatus*	fecal
PP511335	*Bacteroides_H*	93.1	*Eulemur coronatus*	fecal
PP511338	*Barnesiella*	96.9	*Eulemur coronatus*	fecal
PP511338	UMGS27	95.3	*Eulemur coronatus*	fecal
PP511338	*Phocaeicola_A*	90.9	*Eulemur coronatus*	fecal
PP511339	*Phocaeicola*	99.6	*Eulemur coronatus*	fecal
PP511339	*Bacteroides*	99.4	*Eulemur coronatus*	fecal
PP511340	*Bacteroides*	98.5	*Eulemur coronatus*	fecal
PP511340	*Parabacteroides*	97.6	*Eulemur coronatus*	fecal
PP511340	*Mediterranea*	93.2	*Eulemur coronatus*	fecal
PP511341	*Phocaeicola*	99.1	*Eulemur coronatus*	fecal

PP511341	*Bacteroides*	98.5	*Eulemur coronatus*	fecal
PP511343	*Phascolarctobacterium_A*	97.6	*Eulemur coronatus*	fecal
PP511343	CAG-238	97	*Eulemur coronatus*	fecal
PP511352	*Dysosmobacter*	92	*Eulemur coronatus*	fecal
PP511362	*Dysosmobacter*	90	*Eulemur coronatus*	fecal
PP511366	*Mailhella*	91.7	*Eulemur coronatus*	fecal
PP511367	ER4	98.5	*Eulemur coronatus*	fecal
PP511370	*Parabacteroides*	98.7	*Eulemur coronatus*	fecal
PP511372	HGM16780	96.7	*Eulemur coronatus*	fecal
PP511374	*Parabacteroides*	98.7	*Eulemur coronatus*	fecal
PP511376	*Duodenibacillus*	94.5	*Eulemur coronatus*	fecal
PP511434	ER4	98.5	*Eulemur collaris*	fecal
PP511435	*Parabacteroides*	99.1	*Eulemur collaris*	fecal
PP511436	*Parabacteroides*	98.7	*Eulemur collaris*	fecal
PP511439	HGM16780	96.7	*Eulemur collaris*	fecal
PP511382	*Odoribacter*	99.1	*Eulemur collaris*	fecal
PP511384	*Phocaeicola*	97.5	*Eulemur collaris*	fecal
PP511384	*Bacteroides*	97	*Eulemur collaris*	fecal
PP511385	*Bacteroides*	98.4	*Eulemur collaris*	fecal
PP511387	*Bacteroides*	98.7	*Eulemur collaris*	fecal
PP511387	*Phocaeicola*	98.5	*Eulemur collaris*	fecal
PP511388	*Treponema_D*	98.7	*Eulemur collaris*	fecal
PP511390	*Phocaeicola*	99.1	*Eulemur collaris*	fecal
PP511390	*Bacteroides*	98.7	*Eulemur collaris*	fecal
PP511391	*Bacteroides*	99.6	*Eulemur collaris*	fecal
PP511391	*Phocaeicola*	99.6	*Eulemur collaris*	fecal
PP511391	*Bacteroides_G*	90.7	*Eulemur collaris*	fecal
PP511392	*Bacteroides*	99.7	*Eulemur collaris*	fecal
PP511392	*Phocaeicola*	99.6	*Eulemur collaris*	fecal
PP511396	*Bacteroides*	99.6	*Eulemur collaris*	fecal
PP511396	*Phocaeicola*	99.6	*Eulemur collaris*	fecal
PP511396	*Bacteroides_G*	94.9	*Eulemur collaris*	fecal
PP511396	*Bacteroides_I*	93.5	*Eulemur collaris*	fecal
PP511397	*Barnesiella*	95.7	*Eulemur collaris*	fecal
PP511397	UMGS27	93.4	*Eulemur collaris*	fecal
PP511398	*Alistipes*	98.4	*Eulemur collaris*	fecal
PP511399	*Parabacteroides*	97.2	*Eulemur collaris*	fecal
PP511400	*Bacteroides*	98.5	*Eulemur collaris*	fecal
PP511400	*Parabacteroides*	97.6	*Eulemur collaris*	fecal
PP511400	*Mediterranea*	93.2	*Eulemur collaris*	fecal
PP511402	*Phocaeicola*	99.1	*Eulemur collaris*	fecal
PP511402	*Bacteroides*	98.7	*Eulemur collaris*	fecal
PP511403	*Phocaeicola*	99.7	*Eulemur collaris*	fecal
PP511403	*Bacteroides*	99.6	*Eulemur collaris*	fecal
PP511403	*Mediterranea*	99.6	*Eulemur collaris*	fecal
PP511404	*Alistipes*	93.6	*Eulemur collaris*	fecal

PP511405	CAKQMD01	97.9	*Eulemur collaris*	fecal
PP511405	*Scatacola_A*	91.6	*Eulemur collaris*	fecal
PP511407	*Bacteroides*	98.5	*Eulemur collaris*	fecal
PP511407	*Phocaeicola*	98.5	*Eulemur collaris*	fecal
PP511413	*Alistipes_A*	90.2	*Eulemur collaris*	fecal
PP511418	*Limivicinus*	98.4	*Eulemur collaris*	fecal
PP511418	*Phascolarctobacterium_A*	91.6	*Eulemur collaris*	fecal
PP511423	*Dysosmobacter*	90	*Eulemur collaris*	fecal
PP511427	*Dysosmobacter*	94.6	*Eulemur collaris*	fecal
PP511429	*Arcticibacter*	92.2	*Eulemur collaris*	fecal
PP511444	*Bacteroides*	92.2	*Propithecus coquereli*	fecal
PP511446	*Phocaeicola*	99.1	*Varecia variegata*	fecal
PP511446	*Bacteroides*	98.7	*Varecia variegata*	fecal
PP511448	*Phocaeicola*	97.8	*Varecia variegata*	fecal
PP511448	*Bacteroides*	97.2	*Varecia variegata*	fecal
PP511449	*Mediterranea*	95.9	*Varecia variegata*	fecal
PP511449	*Bacteroides*	94.3	*Varecia variegata*	fecal
PP511450	*Bacteroides*	97.8	*Varecia variegata*	fecal
PP511452	*Phocaeicola*	99	*Varecia variegata*	fecal
PP511452	*Bacteroides*	98.7	*Varecia variegata*	fecal
PP511453	*Bacteroides*	99.6	*Varecia variegata*	fecal
PP511453	*Phocaeicola*	99.3	*Varecia variegata*	fecal
PP511454	*Bacteroides*	99.7	*Varecia variegata*	fecal
PP511454	*Phocaeicola*	99.7	*Varecia variegata*	fecal
PP511457	*Barnesiella*	96.9	*Varecia variegata*	fecal
PP511457	UMGS27	95.3	*Varecia variegata*	fecal
PP511457	*Phocaeicola_A*	90.9	*Varecia variegata*	fecal
PP511458	*Phocaeicola*	99.6	*Varecia variegata*	fecal
PP511458	*Bacteroides*	99.4	*Varecia variegata*	fecal
PP511459	*Bacteroides*	98.5	*Varecia variegata*	fecal
PP511459	*Parabacteroides*	97.6	*Varecia variegata*	fecal
PP511459	*Mediterranea*	93.2	*Varecia variegata*	fecal
PP511460	*Phocaeicola*	99.1	*Varecia variegata*	fecal
PP511460	*Bacteroides*	98.5	*Varecia variegata*	fecal
PP511462	*Phascolarctobacterium_A*	97.6	*Varecia variegata*	fecal
PP511462	CAG-238	97	*Varecia variegata*	fecal
PP511466	*Dysosmobacter*	92	*Varecia variegata*	fecal
PP511474	*Dysosmobacter*	90	*Varecia variegata*	fecal
PP511477	ER4	98.5	*Varecia variegata*	fecal
PP511479	*Parabacteroides*	98.7	*Varecia variegata*	fecal
PP511480	*Duodenibacillus*	94.5	*Varecia variegata*	fecal
PP511496	*Alistipes*	93.6	*Varecia variegata*	fecal
PP511498	*Bacteroides*	98.5	*Varecia variegata*	fecal
PP511498	*Phocaeicola*	98.5	*Varecia variegata*	fecal
PP511508	*Succinivibrio*	91.6	*Varecia variegata*	fecal
PP511509	*Arcticibacter*	92.2	*Varecia variegata*	fecal

PP511513	*Parabacteroides*	99.3	*Varecia variegata*	fecal
PP511484	*Phocaeicola*	97.6	*Varecia variegata*	fecal
PP511484	*Bacteroides*	97	*Varecia variegata*	fecal
PP511485	*Bacteroides*	99.6	*Varecia variegata*	fecal
PP511485	*Phocaeicola*	99.6	*Varecia variegata*	fecal
PP511486	*Treponema_D*	98.7	*Varecia variegata*	fecal
PP511488	*Phocaeicola*	99.1	*Varecia variegata*	fecal
PP511488	*Bacteroides*	98.7	*Varecia variegata*	fecal
PP511489	*Bacteroides*	99.6	*Varecia variegata*	fecal
PP511489	*Phocaeicola*	99.4	*Varecia variegata*	fecal
PP511489	*Bacteroides_G*	94.5	*Varecia variegata*	fecal
PP511489	*Bacteroides_I*	92.2	*Varecia variegata*	fecal
PP511490	*Alistipes*	98.5	*Varecia variegata*	fecal
PP511491	*Alistipes*	97.8	*Varecia variegata*	fecal
PP511492	*Phocaeicola*	99.6	*Varecia variegata*	fecal
PP511492	*Bacteroides*	99.4	*Varecia variegata*	fecal
PP511494	*Phocaeicola*	99.1	*Varecia variegata*	fecal
PP511494	*Bacteroides*	98.7	*Varecia variegata*	fecal
PP511495	*Phocaeicola*	99.7	*Varecia variegata*	fecal
PP511495	*Bacteroides*	99.6	*Varecia variegata*	fecal
PP511495	*Mediterranea*	99.6	*Varecia variegata*	fecal
PP511571	*Dysosmobacter*	90	*Varecia variegata*	fecal
PP511574	*Alistipes_A*	93.6	*Varecia variegata*	fecal
PP511575	*Dysosmobacter*	94.6	*Varecia variegata*	fecal
PP511577	ER4	95.6	*Varecia variegata*	fecal
PP511582	ER4	98.5	*Varecia variegata*	fecal
PP511583	*Parasutterella*	99.6	*Varecia variegata*	fecal
PP511584	*Parabacteroides*	98.7	*Varecia variegata*	fecal
PP511587	HGM16780	96.7	*Varecia variegata*	fecal
PP511590	*Phascolarctobacterium_A*	96	*Varecia variegata*	fecal
PP511524	*Phocaeicola*	97.5	*Varecia variegata*	fecal
PP511524	*Bacteroides*	97	*Varecia variegata*	fecal
PP511526	*Bacteroides*	99.7	*Varecia variegata*	fecal
PP511526	*Phocaeicola*	99.7	*Varecia variegata*	fecal
PP511526	*Murdochiella*	91	*Varecia variegata*	fecal
PP511528	*Phocaeicola*	99.1	*Varecia variegata*	fecal
PP511528	*Bacteroides*	98.7	*Varecia variegata*	fecal
PP511529	*Bacteroides*	99.6	*Varecia variegata*	fecal
PP511529	*Phocaeicola*	99.6	*Varecia variegata*	fecal
PP511530	*Bacteroides*	99.6	*Varecia variegata*	fecal
PP511530	*Phocaeicola*	99.3	*Varecia variegata*	fecal
PP511531	*Bacteroides*	99.6	*Varecia variegata*	fecal
PP511531	*Phocaeicola*	99.6	*Varecia variegata*	fecal
PP511531	*Mediterranea*	99.3	*Varecia variegata*	fecal
PP511533	UBA7173	90.9	*Varecia variegata*	fecal
PP511534	*Bacteroides*	99.6	*Varecia variegata*	fecal

PP511534	*Phocaeicola*	99.6	*Varecia variegata*	fecal
PP511534	*Bacteroides_G*	95.3	*Varecia variegata*	fecal
PP511534	*Bacteroides_I*	94.5	*Varecia variegata*	fecal
PP511535	*Bacteroides*	98.5	*Varecia variegata*	fecal
PP511535	*Mediterranea*	97.5	*Varecia variegata*	fecal
PP511535	*Phocaeicola*	97.5	*Varecia variegata*	fecal
PP511535	*Parabacteroides*	93.6	*Varecia variegata*	fecal
PP511536	*Alistipes*	98.4	*Varecia variegata*	fecal
PP511537	*Bacteroides*	98.4	*Varecia variegata*	fecal
PP511537	*Parabacteroides*	95.4	*Varecia variegata*	fecal
PP511537	*Mediterranea*	90.7	*Varecia variegata*	fecal
PP511538	*Parabacteroides*	96.9	*Varecia variegata*	fecal
PP511540	*Phocaeicola*	99.1	*Varecia variegata*	fecal
PP511540	*Bacteroides*	98.7	*Varecia variegata*	fecal
PP511541	*Phocaeicola*	99.7	*Varecia variegata*	fecal
PP511541	*Bacteroides*	99.6	*Varecia variegata*	fecal
PP511541	*Mediterranea*	99.6	*Varecia variegata*	fecal
PP511542	*Alistipes*	93.6	*Varecia variegata*	fecal
PP511546	*Faecalibacterium*	91	*Varecia variegata*	fecal
PP511547	*Bacteroides*	98.5	*Varecia variegata*	fecal
PP511547	*Phocaeicola*	98.5	*Varecia variegata*	fecal
PP511550	*Dysosmobacter*	96.9	*Varecia variegata*	fecal
PP511550	ER4	96.6	*Varecia variegata*	fecal
PP511553	ER4	98.1	*Varecia variegata*	fecal
PP511554	*Phascolarctobacterium_A*	95.6	*Varecia variegata*	fecal
PP511566	ER4	98.4	*Varecia variegata*	fecal
PP511598	*Phocaeicola*	97.5	*Varecia variegata*	oral
PP511598	*Bacteroides*	97	*Varecia variegata*	oral
PP511599	*Treponema_D*	98.7	*Varecia variegata*	oral
PP511600	*Phocaeicola*	99.1	*Varecia variegata*	oral
PP511600	*Bacteroides*	98.7	*Varecia variegata*	oral
PP511601	*Bacteroides*	99.6	*Varecia variegata*	oral
PP511601	*Phocaeicola*	99.6	*Varecia variegata*	oral
PP511602	*Bacteroides*	99.6	*Varecia variegata*	oral
PP511602	*Phocaeicola*	99.6	*Varecia variegata*	oral
PP511602	*Bacteroides_G*	94.9	*Varecia variegata*	oral
PP511602	*Bacteroides_I*	94.5	*Varecia variegata*	oral
PP511603	*Phocaeicola*	99.1	*Varecia variegata*	oral
PP511603	*Bacteroides*	98.7	*Varecia variegata*	oral
PP511604	CAKQMD01	97.9	*Varecia variegata*	oral
PP511604	*Scatacola_A*	91.6	*Varecia variegata*	oral
PP511605	*Bacteroides*	98.5	*Varecia variegata*	oral
PP511605	*Phocaeicola*	98.5	*Varecia variegata*	oral
PP511612	*Bacteroides_I*	93.5	*Varecia variegata*	oral
PP511613	*Bacteroides*	93.5	*Varecia variegata*	oral
PP511618	*Bacteroides*	99.6	*Varecia variegata*	fecal

PP511618	*Phocaeicola*	99.4	*Varecia variegata*	fecal
PP511618	*Bacteroides_G*	94.9	*Varecia variegata*	fecal
PP511618	*Bacteroides_I*	93.5	*Varecia variegata*	fecal
PP511619	*Parabacteroides*	97.2	*Varecia variegata*	fecal
PP511620	*Bacteroides*	98.2	*Varecia variegata*	fecal
PP511620	*Parabacteroides*	96.4	*Varecia variegata*	fecal
PP511621	*Phocaeicola*	99.1	*Varecia variegata*	fecal
PP511621	*Bacteroides*	98.7	*Varecia variegata*	fecal
PP511622	*Phocaeicola*	99.7	*Varecia variegata*	fecal
PP511622	*Bacteroides*	99.6	*Varecia variegata*	fecal
PP511622	*Mediterranea*	99.6	*Varecia variegata*	fecal
PP511624	*Bacteroides*	98.5	*Varecia variegata*	fecal
PP511624	*Phocaeicola*	98.5	*Varecia variegata*	fecal
PP511630	*Limivicinus*	94.6	*Varecia variegata*	fecal
PP511633	*Succinivibrio*	91.6	*Varecia variegata*	fecal
PP511634	*Arcticibacter*	92.2	*Varecia variegata*	fecal
PP511635	*Dysosmobacter*	90	*Varecia variegata*	fecal
PP511637	ER4	98.5	*Varecia variegata*	fecal
PP511638	*Parabacteroides*	99.3	*Varecia variegata*	fecal
PP511615	*Bacteroides*	99.6	*Varecia variegata*	fecal
PP511615	*Phocaeicola*	99.6	*Varecia variegata*	fecal
PP511617	*Phocaeicola*	99.1	*Varecia variegata*	fecal
PP511617	*Bacteroides*	98.7	*Varecia variegata*	fecal
PP511670	*Alistipes_A*	90.2	*Varecia variegata*	fecal
PP511674	*Limivicinus*	97.9	*Varecia variegata*	fecal
PP511676	*Dysosmobacter*	90	*Varecia variegata*	fecal
PP511679	*Dysosmobacter*	94.6	*Varecia variegata*	fecal
PP511680	*Arcticibacter*	92.2	*Varecia variegata*	fecal
PP511682	ER4	98.5	*Varecia variegata*	fecal
PP511685	HGM16780	96.7	*Varecia variegata*	fecal
PP511643	*Odoribacter*	99.1	*Varecia variegata*	fecal
PP511644	*Phocaeicola*	97.5	*Varecia variegata*	fecal
PP511644	*Bacteroides*	97	*Varecia variegata*	fecal
PP511645	*Bacteroides*	98.4	*Varecia variegata*	fecal
PP511646	*Bacteroides*	99.7	*Varecia variegata*	fecal
PP511646	*Phocaeicola*	99.7	*Varecia variegata*	fecal
PP511646	*Murdochiella*	91	*Varecia variegata*	fecal
PP511647	*Bacteroides*	98.7	*Varecia variegata*	fecal
PP511647	*Phocaeicola*	98.5	*Varecia variegata*	fecal
PP511648	*Bacteroides*	99.6	*Varecia variegata*	fecal
PP511648	*Phocaeicola*	99.6	*Varecia variegata*	fecal
PP511649	*Treponema_D*	98.7	*Varecia variegata*	fecal
PP511651	*Phocaeicola*	99.1	*Varecia variegata*	fecal
PP511651	*Bacteroides*	98.7	*Varecia variegata*	fecal
PP511652	*Bacteroides*	99.6	*Varecia variegata*	fecal
PP511652	*Phocaeicola*	99.6	*Varecia variegata*	fecal

PP511653	*Bacteroides*	99.6	*Varecia variegata*	fecal
PP511653	*Phocaeicola*	99.6	*Varecia variegata*	fecal
PP511653	*Mediterranea*	99.3	*Varecia variegata*	fecal
PP511654	*Bacteroides*	99.6	*Varecia variegata*	fecal
PP511654	*Phocaeicola*	99.4	*Varecia variegata*	fecal
PP511654	*Bacteroides_G*	94.9	*Varecia variegata*	fecal
PP511654	*Bacteroides_I*	93.5	*Varecia variegata*	fecal
PP511655	*Barnesiella*	95.7	*Varecia variegata*	fecal
PP511655	UMGS27	93.4	*Varecia variegata*	fecal
PP511656	*Alistipes*	98.4	*Varecia variegata*	fecal
PP511657	*Alistipes*	97.8	*Varecia variegata*	fecal
PP511658	*Bacteroides*	98.2	*Varecia variegata*	fecal
PP511658	*Parabacteroides*	96.3	*Varecia variegata*	fecal
PP511660	*Phocaeicola*	99.1	*Varecia variegata*	fecal
PP511660	*Bacteroides*	98.7	*Varecia variegata*	fecal
PP511661	*Phocaeicola*	99.7	*Varecia variegata*	fecal
PP511661	*Bacteroides*	99.6	*Varecia variegata*	fecal
PP511661	*Mediterranea*	99.6	*Varecia variegata*	fecal
PP511662	CAKQMD01	97.9	*Varecia variegata*	fecal
PP511662	*Scatacola_A*	91.6	*Varecia variegata*	fecal
PP511663	*Alistipes*	93.6	*Varecia variegata*	fecal
PP511665	*Bacteroides*	98.5	*Varecia variegata*	fecal
PP511665	*Phocaeicola*	98.5	*Varecia variegata*	fecal
PP511695	*Bacteroides_I*	93.5	*Varecia variegata*	oral
PP511696	*Bacteroides*	93.5	*Varecia variegata*	oral
PP511703	*Bacteroides_I*	92.6	*Varecia variegata*	oral
PP511704	*Bacteroides*	93.5	*Varecia variegata*	oral
PP511705	JABCPE02	98.5	*Varecia variegata*	oral
PP511705	*Chryseobacterium*	97.2	*Varecia variegata*	oral
PP511764	HGM16780	96.7	*Lemur catta*	fecal
PP511767	*Phascolarctobacterium_A*	96	*Lemur catta*	fecal
PP511708	*Phocaeicola*	97.5	*Lemur catta*	fecal
PP511708	*Bacteroides*	97	*Lemur catta*	fecal
PP511710	*Bacteroides*	99.7	*Lemur catta*	fecal
PP511710	*Phocaeicola*	99.7	*Lemur catta*	fecal
PP511710	*Murdochiella*	91	*Lemur catta*	fecal
PP511711	*Bacteroides*	97.5	*Lemur catta*	fecal
PP511711	*Phocaeicola*	94.7	*Lemur catta*	fecal
PP511713	*Phocaeicola*	99.1	*Lemur catta*	fecal
PP511713	*Bacteroides*	98.7	*Lemur catta*	fecal
PP511714	*Bacteroides*	99.6	*Lemur catta*	fecal
PP511714	*Phocaeicola*	99.6	*Lemur catta*	fecal
PP511715	*Bacteroides*	99.6	*Lemur catta*	fecal
PP511715	*Phocaeicola*	99.6	*Lemur catta*	fecal
PP511716	*Bacteroides*	99.6	*Lemur catta*	fecal
PP511716	*Phocaeicola*	99.6	*Lemur catta*	fecal

PP511716	*Mediterranea*	99.3	*Lemur catta*	fecal
PP511717	UBA7173	90.9	*Lemur catta*	fecal
PP511718	*Alistipes*	98.4	*Lemur catta*	fecal
PP511719	*Parabacteroides*	97.2	*Lemur catta*	fecal
PP511720	*Alistipes*	97.8	*Lemur catta*	fecal
PP511721	*Bacteroides*	98.4	*Lemur catta*	fecal
PP511721	*Parabacteroides*	95.4	*Lemur catta*	fecal
PP511721	*Mediterranea*	90.7	*Lemur catta*	fecal
PP511723	*Phocaeicola*	99.1	*Lemur catta*	fecal
PP511723	*Bacteroides*	98.7	*Lemur catta*	fecal
PP511724	*Phocaeicola*	99.7	*Lemur catta*	fecal
PP511724	*Bacteroides*	99.6	*Lemur catta*	fecal
PP511724	*Mediterranea*	99.6	*Lemur catta*	fecal
PP511725	*Alistipes*	93.6	*Lemur catta*	fecal
PP511728	*Faecalibacterium*	91	*Lemur catta*	fecal
PP511729	*Bacteroides*	98.5	*Lemur catta*	fecal
PP511729	*Phocaeicola*	98.5	*Lemur catta*	fecal
PP511734	*Alistipes_A*	90.2	*Lemur catta*	fecal
PP511736	*Phascolarctobacterium_A*	95.6	*Lemur catta*	fecal
PP511743	*Limivicinus*	92.7	*Lemur catta*	fecal
PP511746	ER4	98.4	*Lemur catta*	fecal
PP511750	*Dysosmobacter*	90	*Lemur catta*	fecal
PP511753	*Dysosmobacter*	93.5	*Lemur catta*	fecal
PP511755	ER4	95.6	*Lemur catta*	fecal
PP511758	ER4	98.5	*Lemur catta*	fecal
PP511759	*Parasutterella*	99.6	*Lemur catta*	fecal
PP511761	*Parabacteroides*	98.7	*Lemur catta*	fecal
PP511775	*Phocaeicola*	97.6	*Propithecus coquereli*	fecal
PP511775	*Bacteroides*	97	*Propithecus coquereli*	fecal
PP511776	*Bacteroides*	99.7	*Propithecus coquereli*	fecal
PP511776	*Phocaeicola*	99.7	*Propithecus coquereli*	fecal
PP511781	*Phascolarctobacterium_A*	96	*Propithecus coquereli*	fecal
PP511784	*Faecalibacterium*	95.7	*Propithecus coquereli*	fecal
PP511789	*Bacteroides*	93.9	*Propithecus coquereli*	fecal
PP511839	ER4	98.5	*Propithecus coquereli*	fecal
PP511840	ER4	96.3	*Propithecus coquereli*	fecal
PP511844	*Parabacteroides*	98.7	*Propithecus coquereli*	fecal
PP511796	*Phocaeicola*	97.5	*Propithecus coquereli*	fecal
PP511796	*Bacteroides*	97	*Propithecus coquereli*	fecal
PP511798	*Bacteroides*	98.2	*Propithecus coquereli*	fecal
PP511798	*Phocaeicola*	95.7	*Propithecus coquereli*	fecal
PP511799	*Bacteroides*	99.7	*Propithecus coquereli*	fecal
PP511799	*Phocaeicola*	99.6	*Propithecus coquereli*	fecal
PP511800	*Treponema_D*	98.7	*Propithecus coquereli*	fecal
PP511801	*Bacteroides*	97.5	*Propithecus coquereli*	fecal
PP511802	*Phocaeicola*	99.1	*Propithecus coquereli*	fecal

PP511802	*Bacteroides*	98.7	*Propithecus coquereli*	fecal
PP511803	*Bacteroides*	99.6	*Propithecus coquereli*	fecal
PP511803	*Phocaeicola*	99.6	*Propithecus coquereli*	fecal
PP511806	*Alistipes*	98.5	*Propithecus coquereli*	fecal
PP511807	CAKQMD01	97.9	*Propithecus coquereli*	fecal
PP511807	*Scatacola_A*	91.6	*Propithecus coquereli*	fecal
PP511814	*Dysosmobacter*	96.9	*Propithecus coquereli*	fecal
PP511814	ER4	96.6	*Propithecus coquereli*	fecal
PP511816	*Phascolarctobacterium_A*	94.7	*Propithecus coquereli*	fecal
PP511816	*Alistipes_A*	91.6	*Propithecus coquereli*	fecal
PP511819	*Dysosmobacter*	91.6	*Propithecus coquereli*	fecal
PP511824	*Limivicinus*	98.4	*Propithecus coquereli*	fecal
PP511824	*Phascolarctobacterium_A*	91.6	*Propithecus coquereli*	fecal
PP511828	ER4	98.5	*Propithecus coquereli*	fecal
PP511832	*Phascolarctobacterium_A*	98.1	*Propithecus coquereli*	fecal
PP511832	ER4	97.6	*Propithecus coquereli*	fecal
PP511851	*Phocaeicola*	97.5	*Varecia variegata*	fecal
PP511851	*Bacteroides*	97	*Varecia variegata*	fecal
PP511852	*Bacteroides*	98.4	*Varecia variegata*	fecal
PP511853	*Bacteroides*	99.7	*Varecia variegata*	fecal
PP511853	*Phocaeicola*	99.7	*Varecia variegata*	fecal
PP511853	*Murdochiella*	91	*Varecia variegata*	fecal
PP511854	*Bacteroides*	98.7	*Varecia variegata*	fecal
PP511854	*Phocaeicola*	98.5	*Varecia variegata*	fecal
PP511855	*Bacteroides*	99.7	*Varecia variegata*	fecal
PP511855	*Phocaeicola*	99.6	*Varecia variegata*	fecal
PP511857	*Phocaeicola*	99.1	*Varecia variegata*	fecal
PP511857	*Bacteroides*	98.7	*Varecia variegata*	fecal
PP511858	*Bacteroides*	99.6	*Varecia variegata*	fecal
PP511858	*Phocaeicola*	99.6	*Varecia variegata*	fecal
PP511859	*Bacteroides*	99.6	*Varecia variegata*	fecal
PP511859	*Phocaeicola*	99.6	*Varecia variegata*	fecal
PP511859	*Mediterranea*	99.3	*Varecia variegata*	fecal
PP511860	*Bacteroides*	99.6	*Varecia variegata*	fecal
PP511860	*Phocaeicola*	99.6	*Varecia variegata*	fecal
PP511860	*Bacteroides_G*	95.3	*Varecia variegata*	fecal
PP511860	*Bacteroides_I*	94.5	*Varecia variegata*	fecal
PP511861	*Bacteroides*	98.2	*Varecia variegata*	fecal
PP511861	*Parabacteroides*	96.3	*Varecia variegata*	fecal
PP511862	*Phocaeicola*	99.7	*Varecia variegata*	fecal
PP511862	*Bacteroides*	99.6	*Varecia variegata*	fecal
PP511862	*Mediterranea*	99.6	*Varecia variegata*	fecal
PP511863	*Bacteroides*	98.5	*Varecia variegata*	fecal
PP511863	*Phocaeicola*	98.5	*Varecia variegata*	fecal
PP511877	*Bacteroides*	92.2	*Propithecus coquereli*	fecal
PP511878	*Faecalibacterium*	98.4	*Propithecus coquereli*	fecal

Table S4. Inovirus iPHoP predictions

Accession No.	Bacterial Host Genus	Confidence (%)	Sample species	Sample type
PP511337	*Ruthenibacterium*	90.9	*Eulemur coronatus*	fecal
PP511461	*Ruthenibacterium*	91.2	*Varecia variegata*	fecal
PP511543	*Ruthenibacterium*	91.1	*Varecia variegata*	fecal
PP511608	*Mesocricetibacter*	91.5	*Varecia variegata*	oral
PP511610	*Acinetobacter*	97	*Varecia variegata*	oral
PP511610	*Moraxella*	90.7	*Varecia variegata*	oral
PP511607	*Neisseria*	90.7	*Varecia variegata*	oral
PP511650	*Enterocloster*	97.8	*Varecia variegata*	fecal
PP511664	*Thomasclavelia*	92.9	*Varecia variegata*	fecal
PP511687	*Rodentibacter*	91.4	*Varecia variegata*	oral
PP511689	*Neisseria*	91.8	*Varecia variegata*	oral
PP511698	*Mesocricetibacter*	91.5	*Varecia variegata*	oral
PP511699	*Aggregatibacter*	94.9	*Varecia variegata*	oral
PP511700	*Acinetobacter*	94.7	*Varecia variegata*	oral
PP511700	*Moraxella*	94.5	*Varecia variegata*	oral
PP511726	*Ruthenibacterium*	91	*Lemur catta*	fecal
PP511856	*Enterocloster*	97.8	*Varecia variegata*	fecal

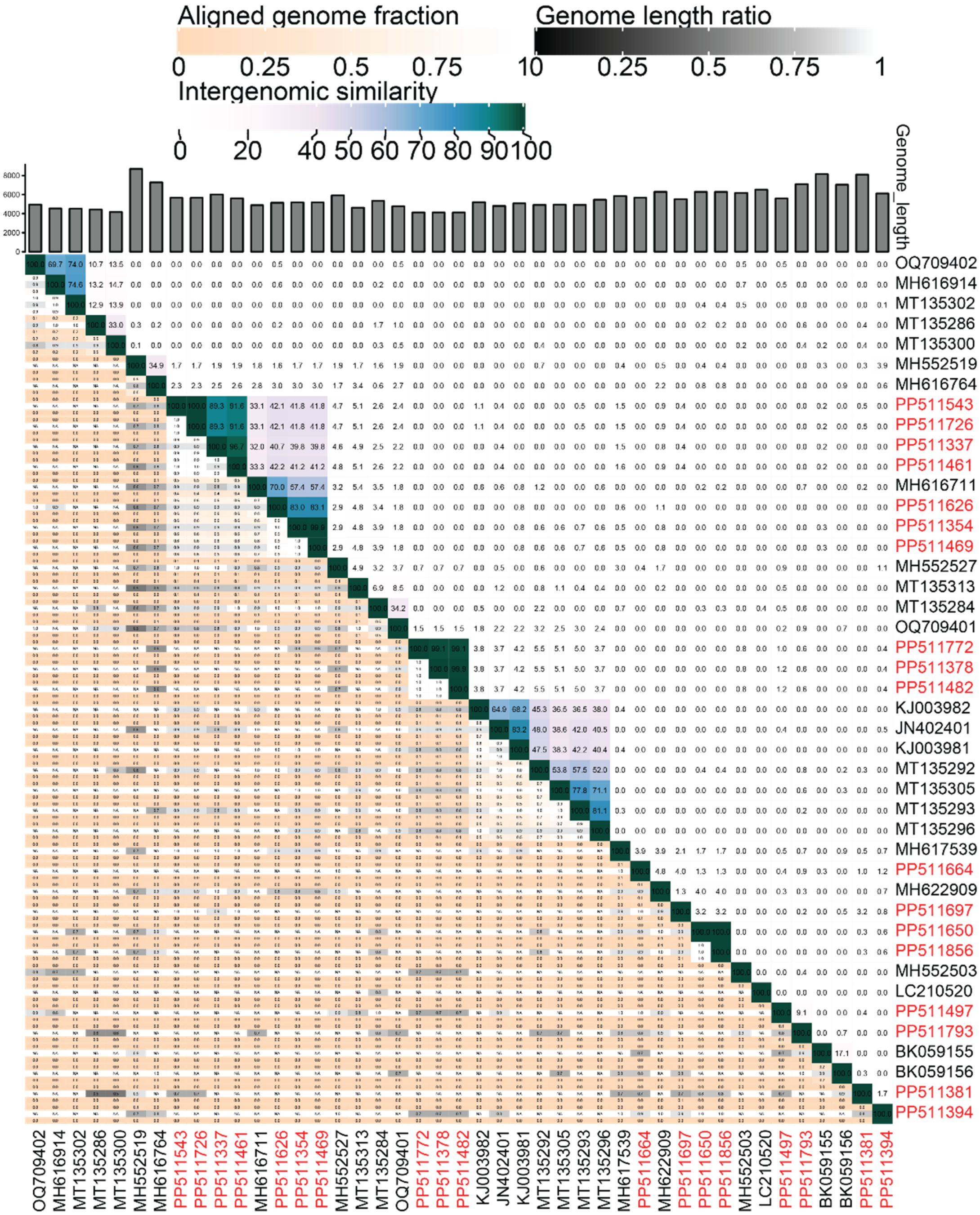
Aligned genome fraction
0
0.25
0.5
0.75
1
Genome length ratio
0
0.25
0.5
0.75
1
Intergenomic similarity
0
20
40
50
60
70
80
90
100
Genome_length
8000
6000
4000
2000
0
OQ709402
MH616914
MT135302
MT135286
MT135300
MH552519
MH616764
PP511543
PP511726
PP511337
PP511461
MH616711
PP511626
PP511354
PP511469
MH552527
MT135313
MT135284
OQ709401
PP511772
PP511378
PP511482
KJ003982
JN402401
KJ003981
MT135292
MT135305
MT135293
MT135296
MH617539
PP511664
MH622909
PP511697
PP511650
PP511856
MH552503
LC210520
PP511497
PP511793
BK059155
BK059156
PP511381
PP511394

Figure S1. VIRIDIC heatmap from a subset of the *Inoviridae* phylogeny of clade 'A' including inoviruses identified in this study.

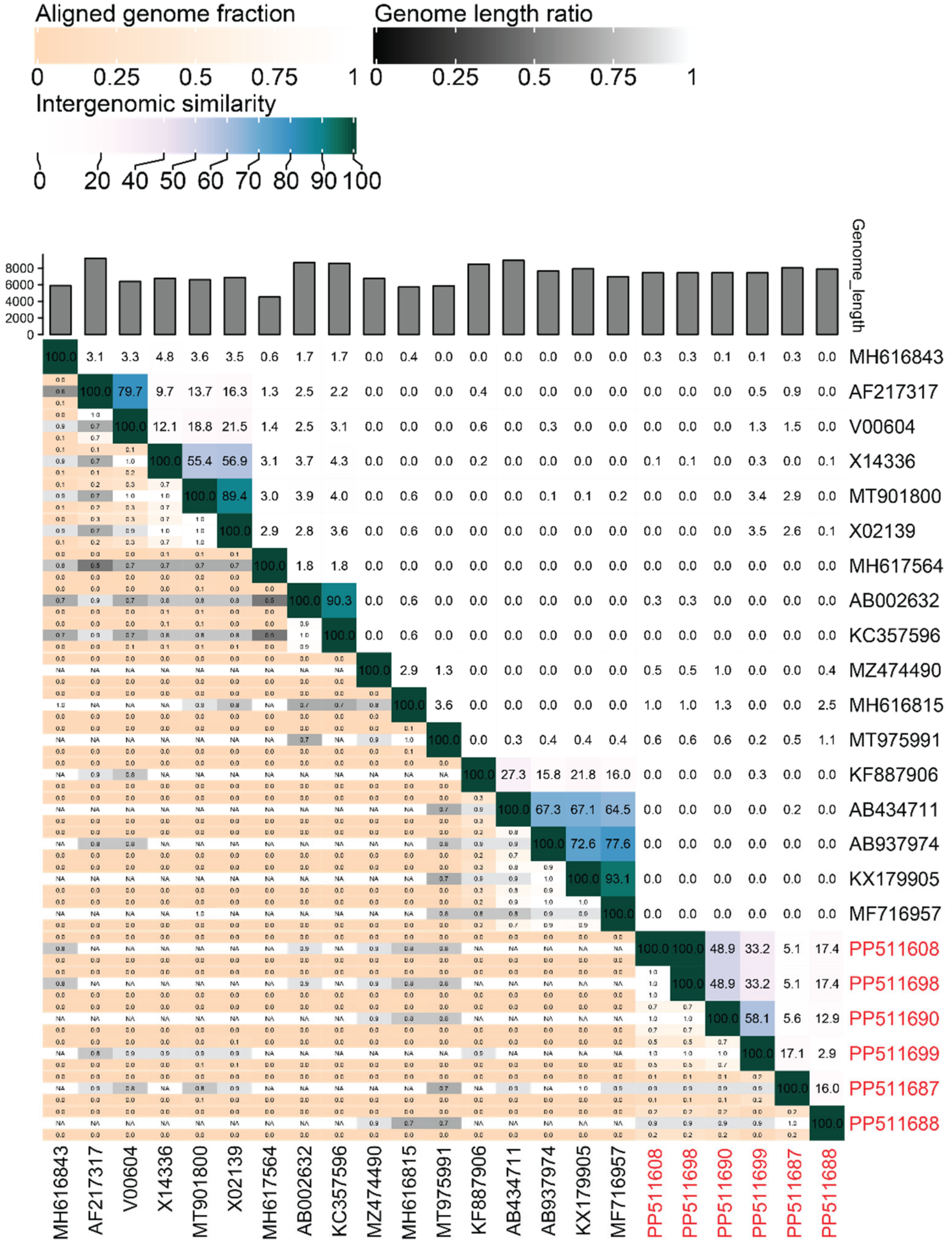

Figure S2. VIRIDIC heatmap from a subset of the *Inoviridae* phylogeny of clade 'B' including inoviruses identified in this study.

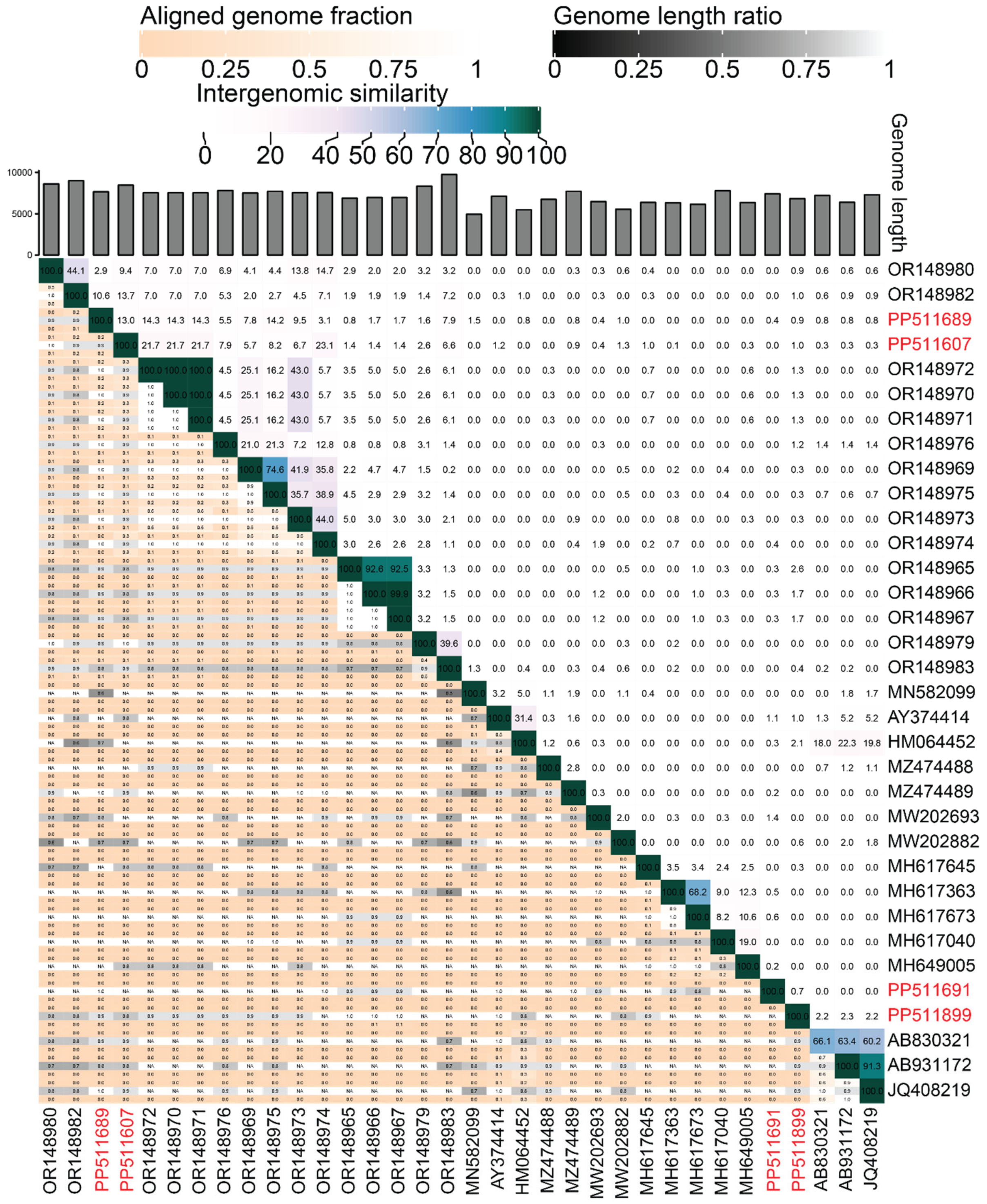

Figure S3. VIRIDIC heatmap from a subset of the *Inoviridae* phylogeny of clade 'C' including inoviruses identified in this study.

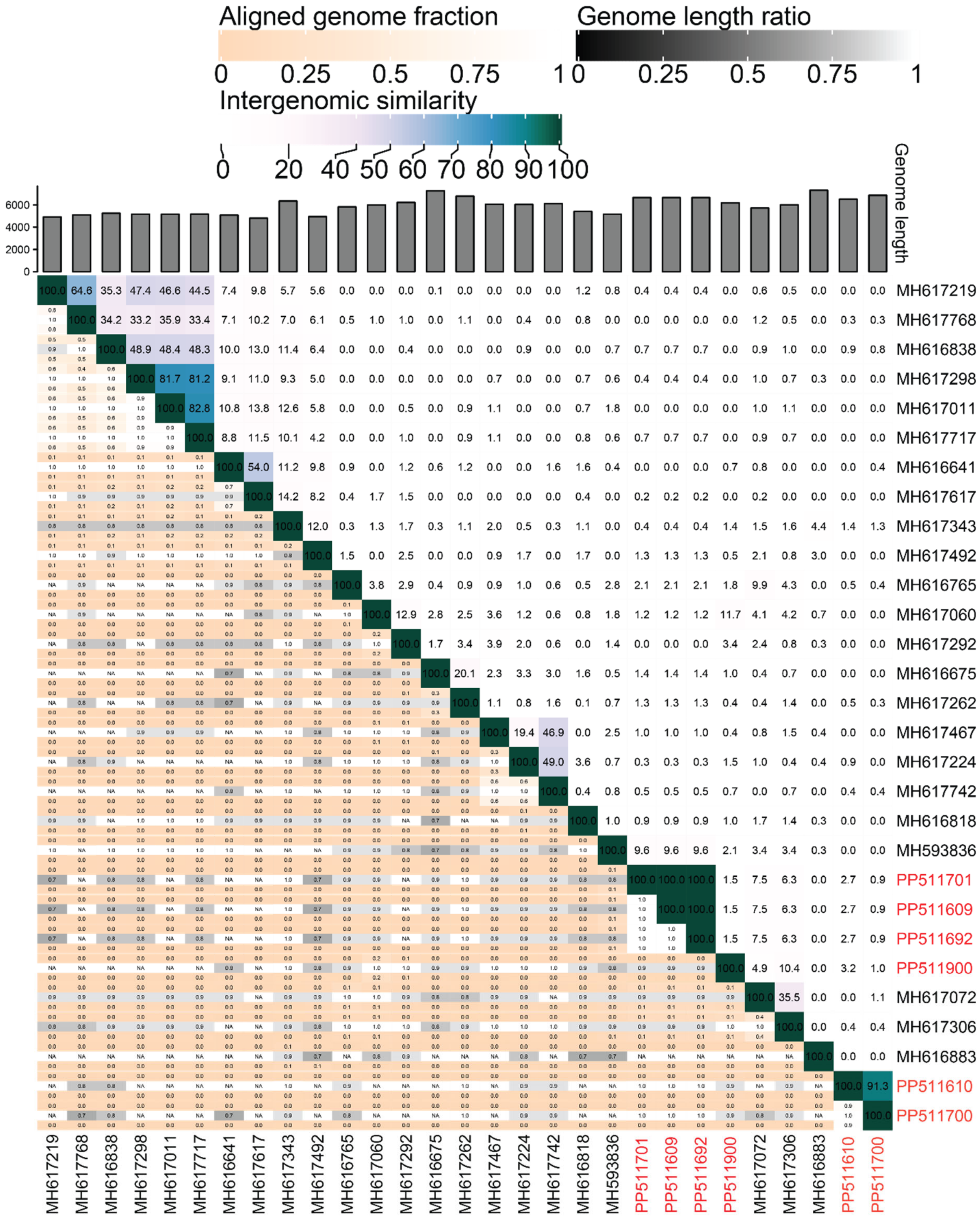

Figure S4. VIRIDIC heatmap from a second subset of the *Inoviridae* phylogeny of clade 'C' including inoviruses identified in this study.

Table S5. Caudovirus iPHoP predictions

Accession No.	Bacterial Host Genus	Confidence (%)	Sample species	Sample type
PP511318	UBA1221	95.3	*Eulemur flavifrons*	fecal
PP511318	*Stercorousia*	91.8	*Eulemur flavifrons*	fecal
PP511379	*Amulumruptor*	97.2	*Eulemur collaris*	fecal
PP511443	*Choladousia*	94.2	*Propithecus coquereli*	fecal
PP511520	*Odoribacter*	95.9	*Varecia variegata*	fecal
PP511520	CAG-302	90.5	*Varecia variegata*	fecal
PP511521	*Massilistercora*	98.6	*Varecia variegata*	fecal
PP511521	*Dorea*	98.1	*Varecia variegata*	fecal
PP511521	*Merdimonas*	98.1	*Varecia variegata*	fecal
PP511521	*Clostridium*	97.5	*Varecia variegata*	fecal
PP511596	*Actinomyces*	98.7	*Varecia variegata*	oral
PP511706	*Sarcina*	93.7	*Lemur catta*	fecal
PP511791	*Frisingicoccus*	91.9	*Propithecus coquereli*	fecal
PP511792	*Enterenecus*	91.1	*Propithecus coquereli*	fecal

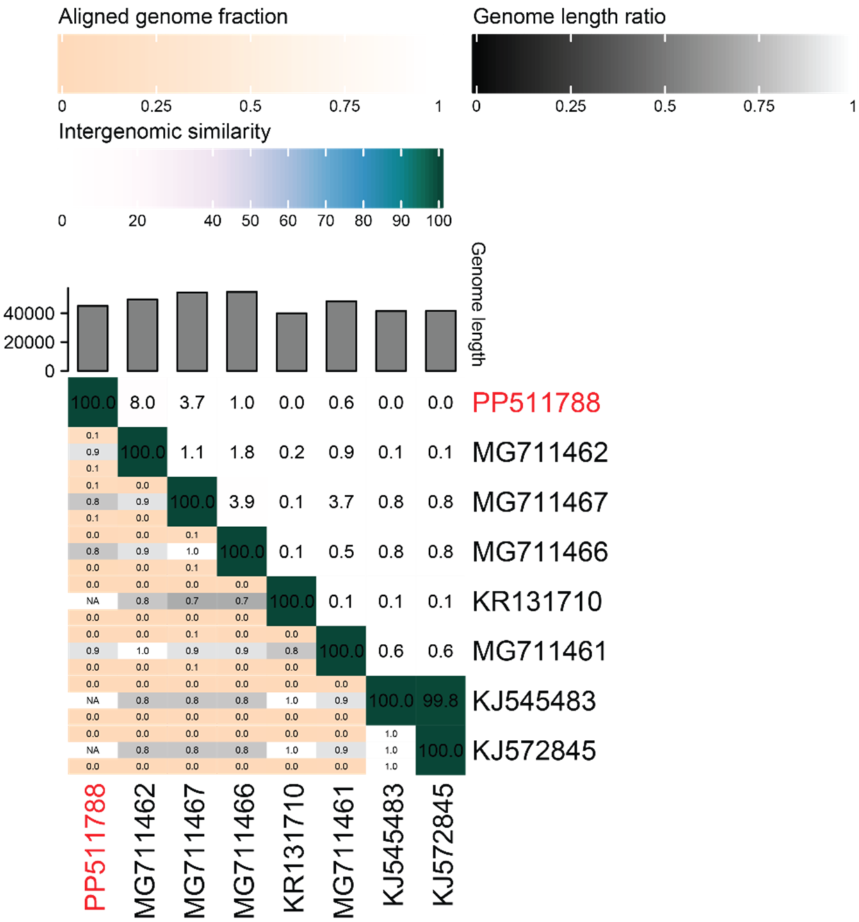

Figure S5. VIRIDIC heatmap from a subset of the caudovirus phylogeny from Figure 18A.

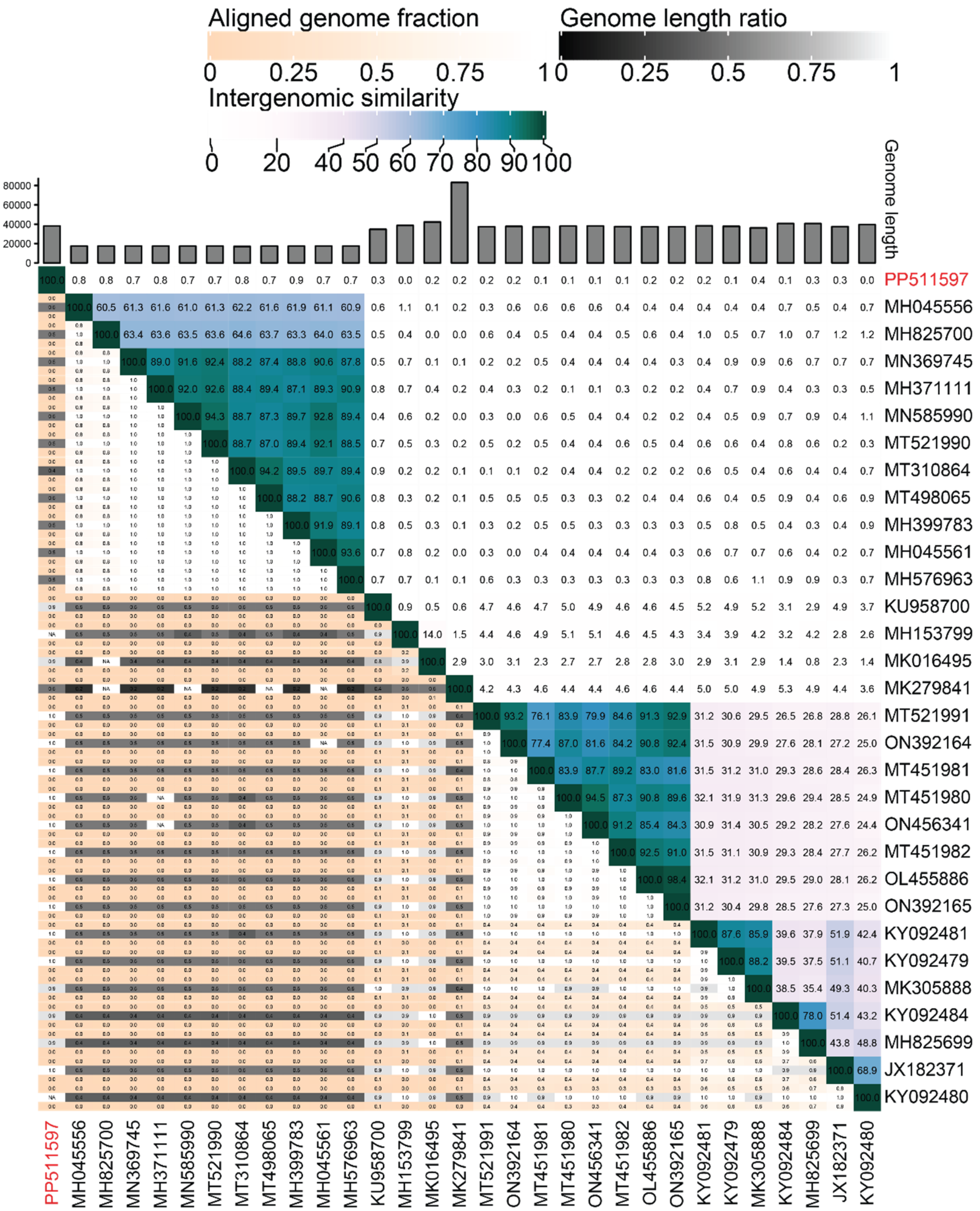

Figure S6. VIRIDIC heatmap from a subset of the caudovirus phylogeny from Figure 18B.

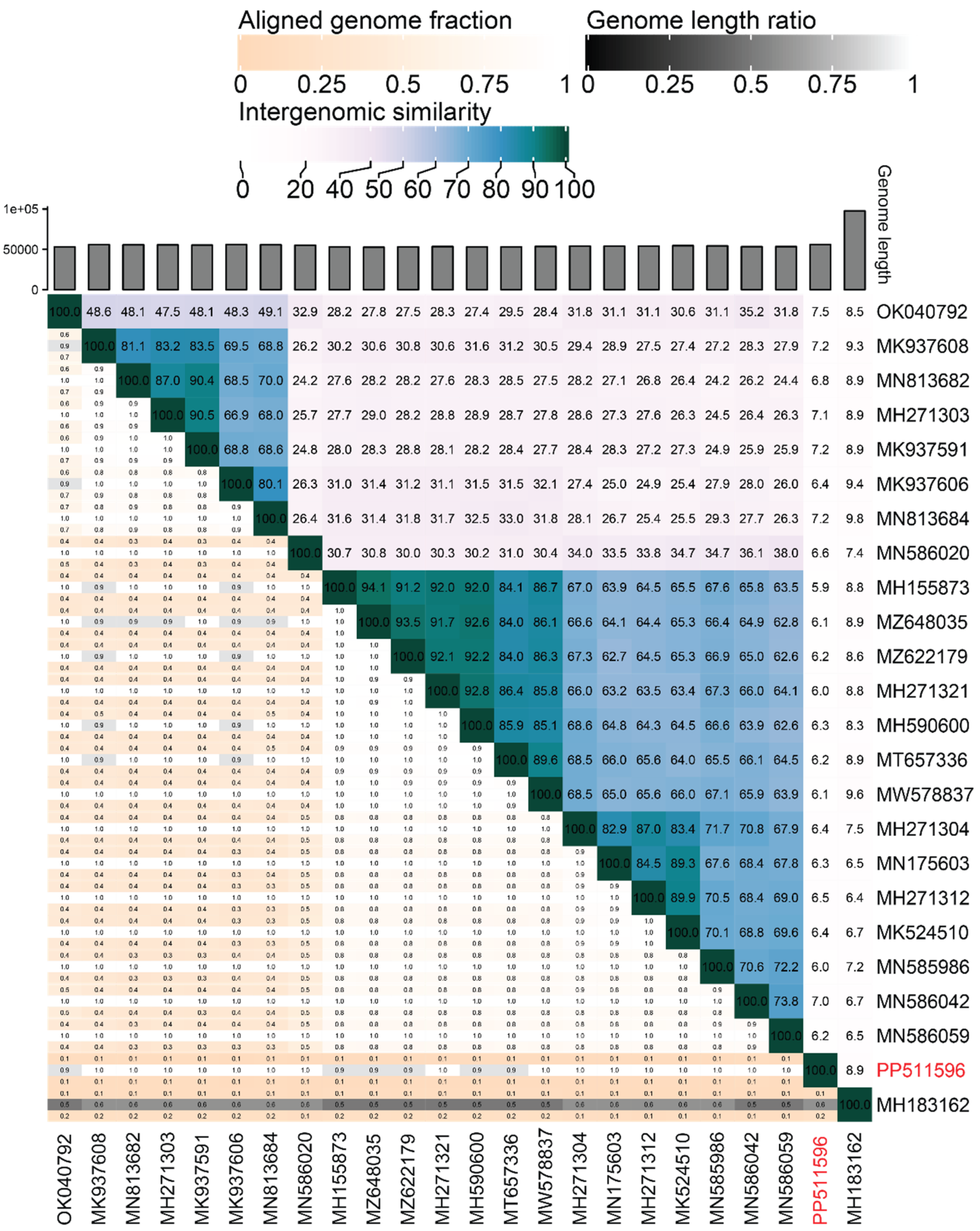

Figure S7. VIRIDIC heatmap from a subset of the caudovirus phylogeny from Figure 18C.

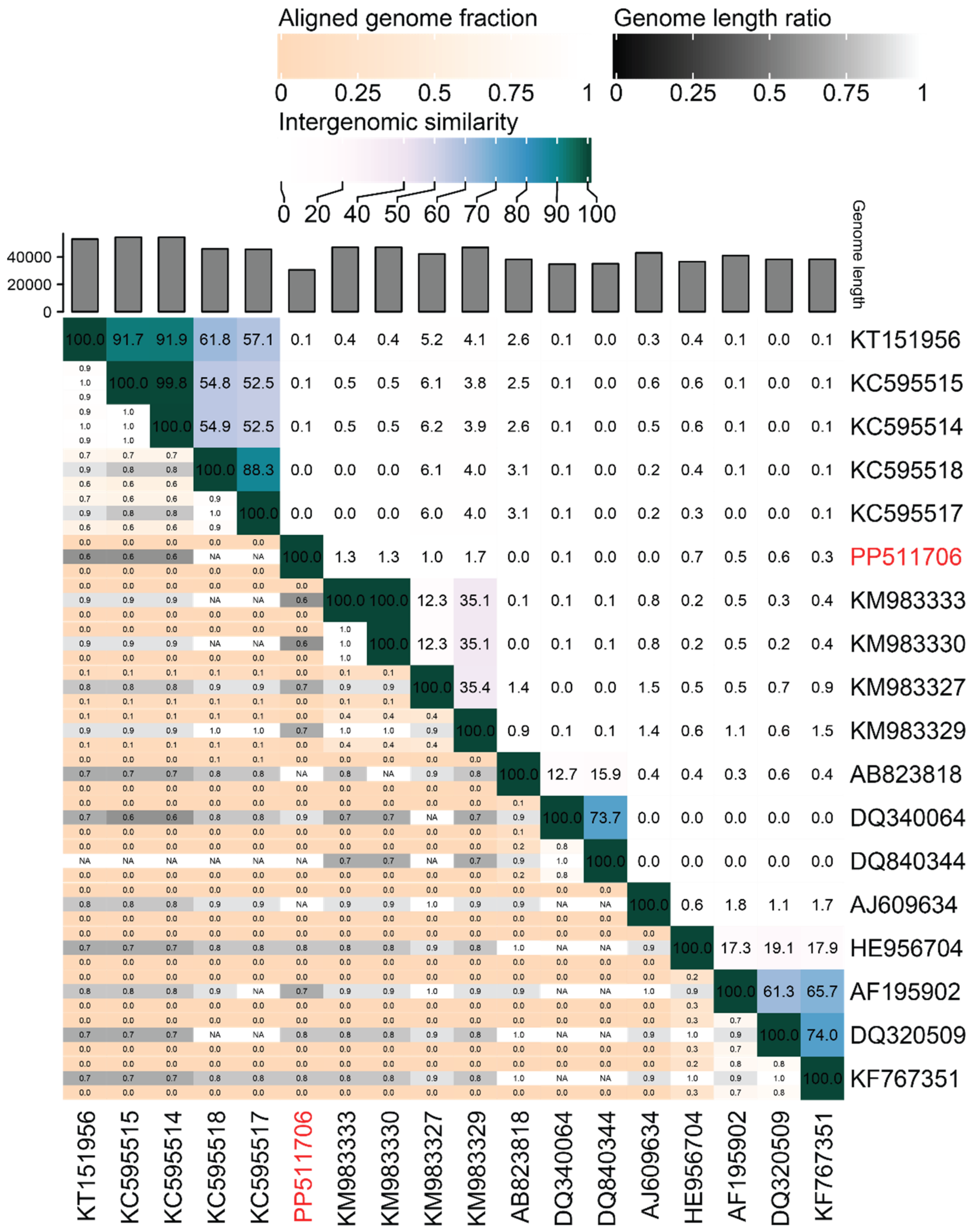

Figure S8. VIRIDIC heatmap from a subset of the caudovirus phylogeny from Figure 18D.

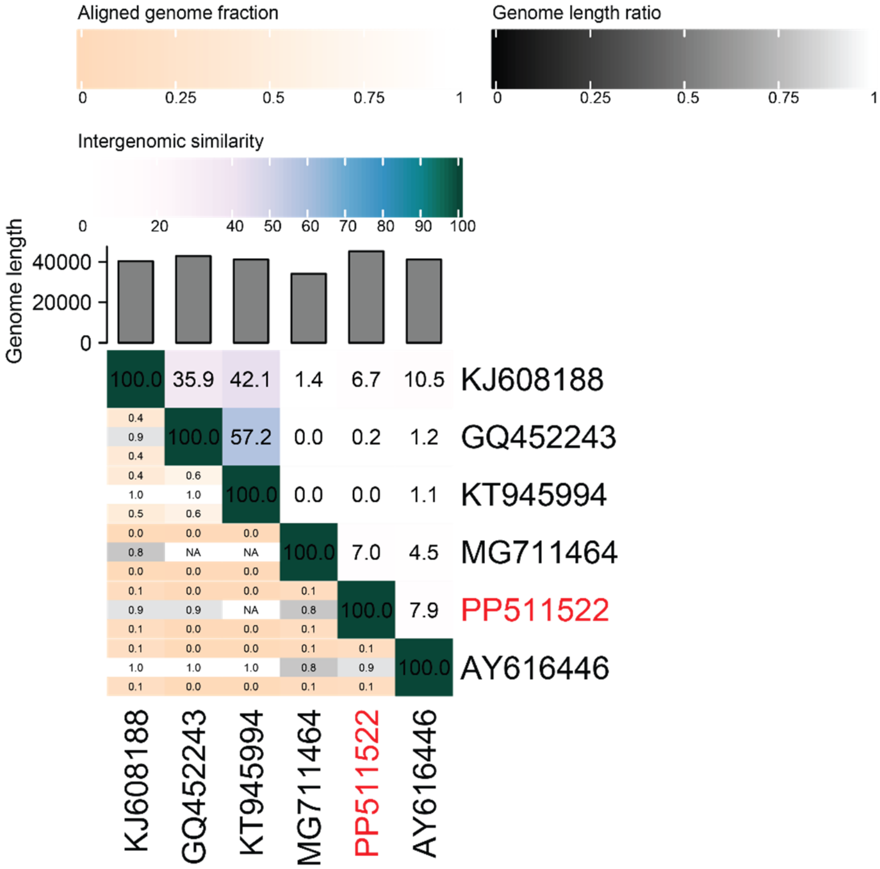

Figure S9. VIRIDIC heatmap from a subset of the caudovirus phylogeny from Figure 18E.

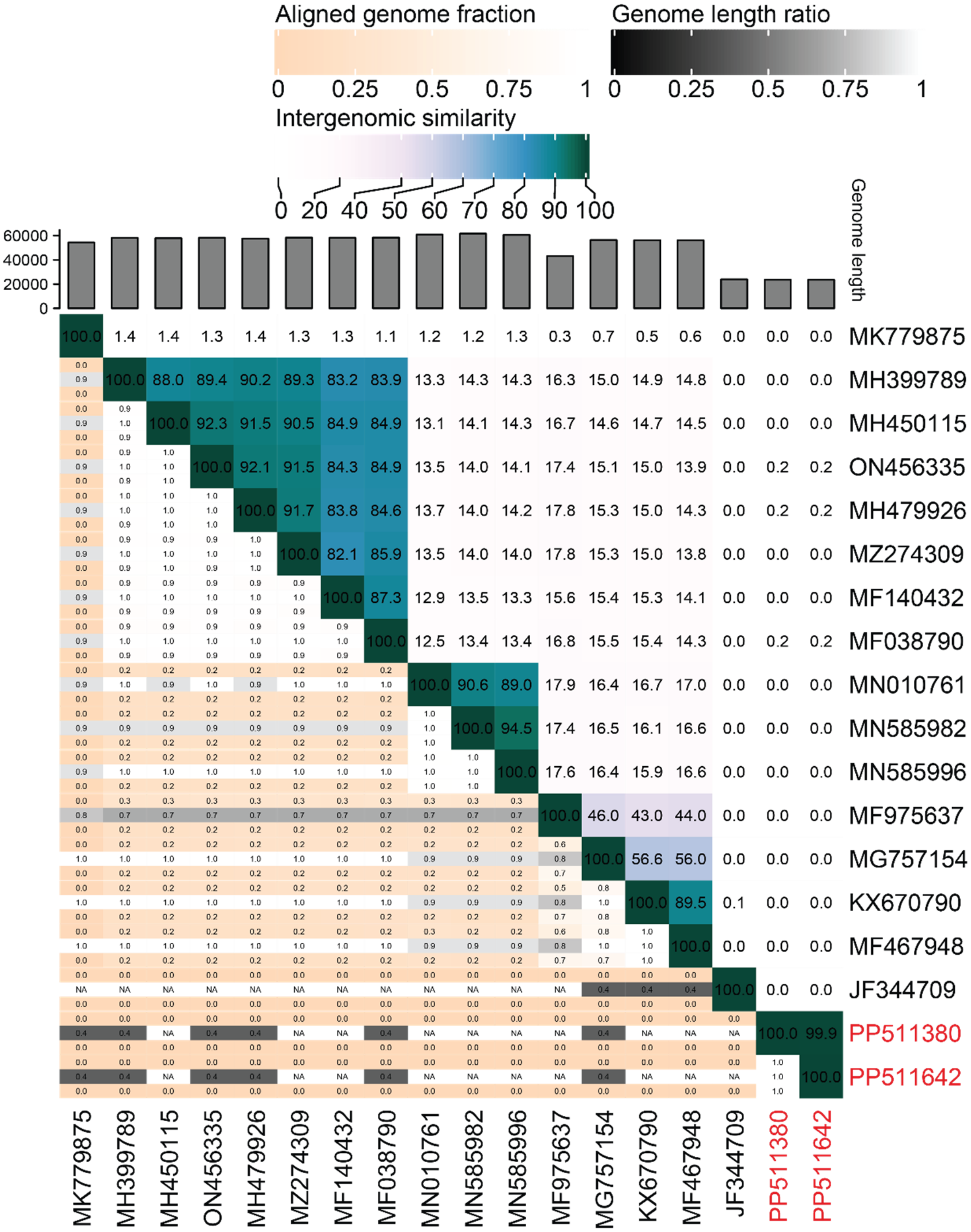

Figure S10. VIRIDIC heatmap from a subset of the caudovirus phylogeny from Figure 18F.

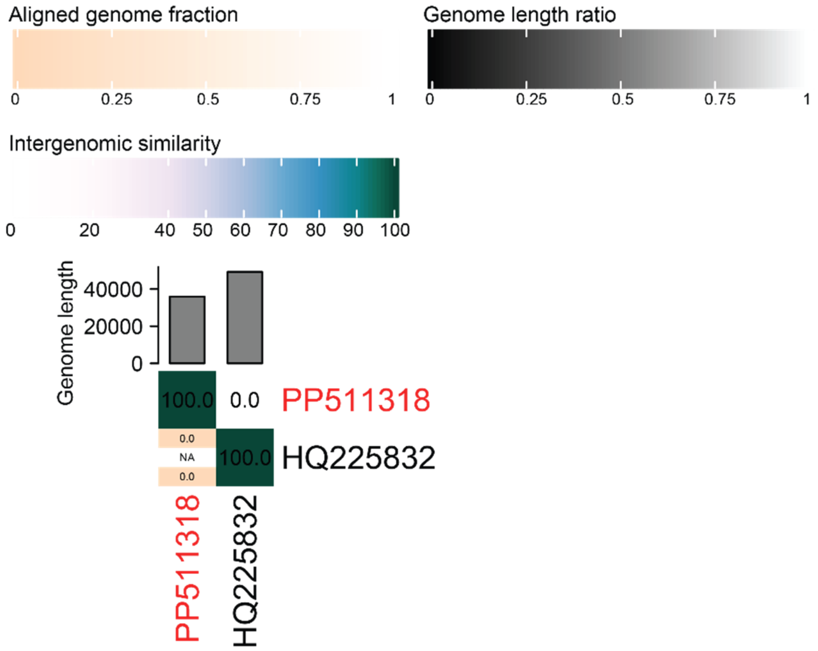

Figure S11. VIRIDIC heatmap from a subset of the caudovirus phylogeny from Figure 18G.

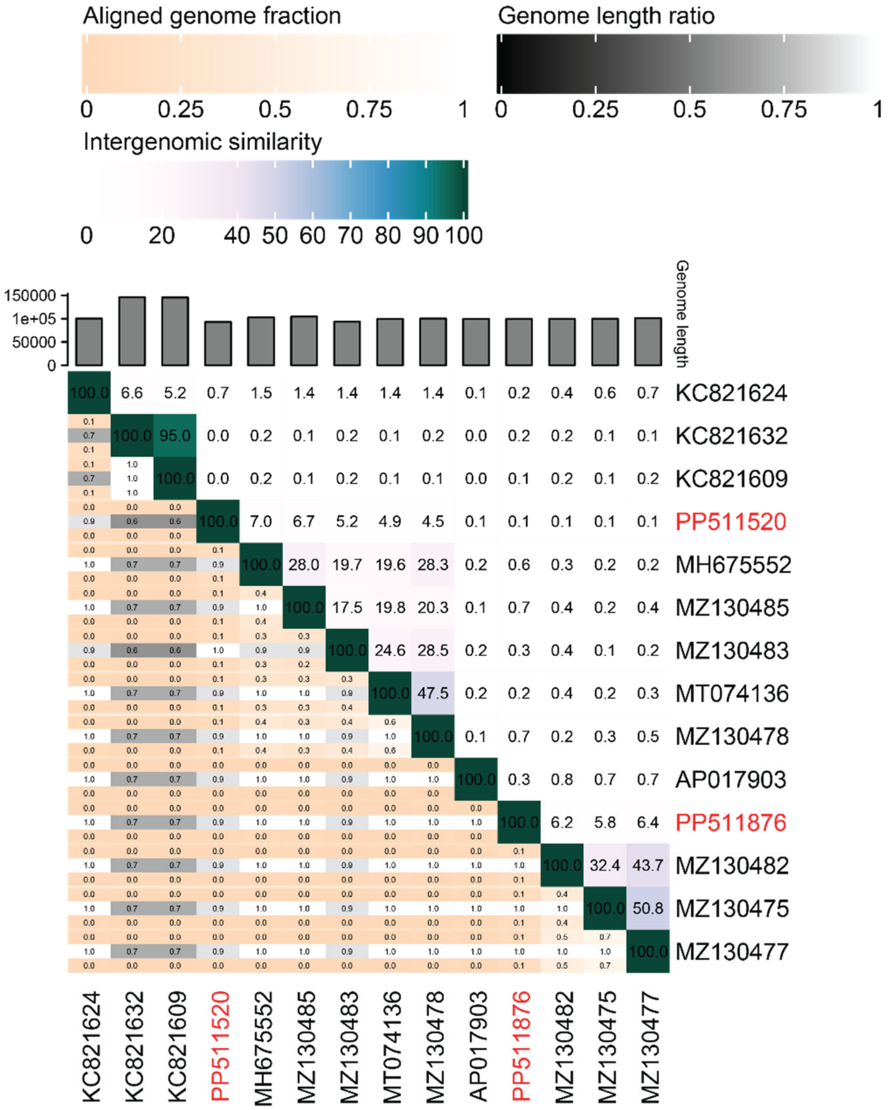

Figure S12. VIRIDIC heatmap from a subset of the caudovirus phylogeny from Figure 18H.

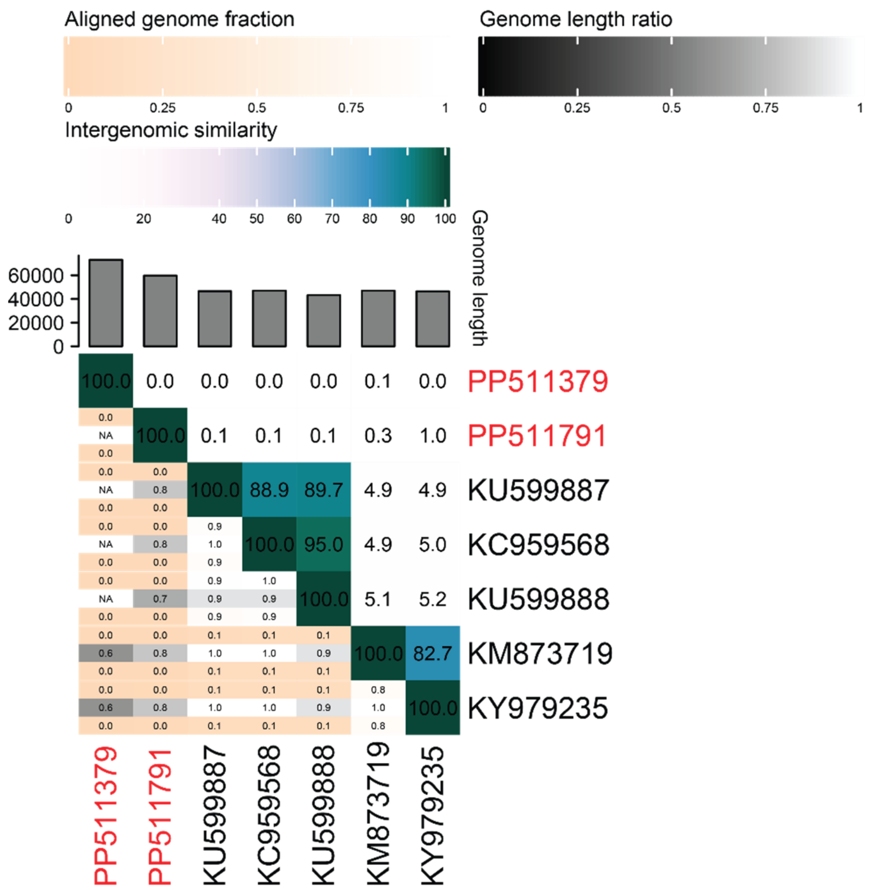

Figure S13. VIRIDIC heatmap from a subset of the caudovirus phylogeny from Figure 18I.

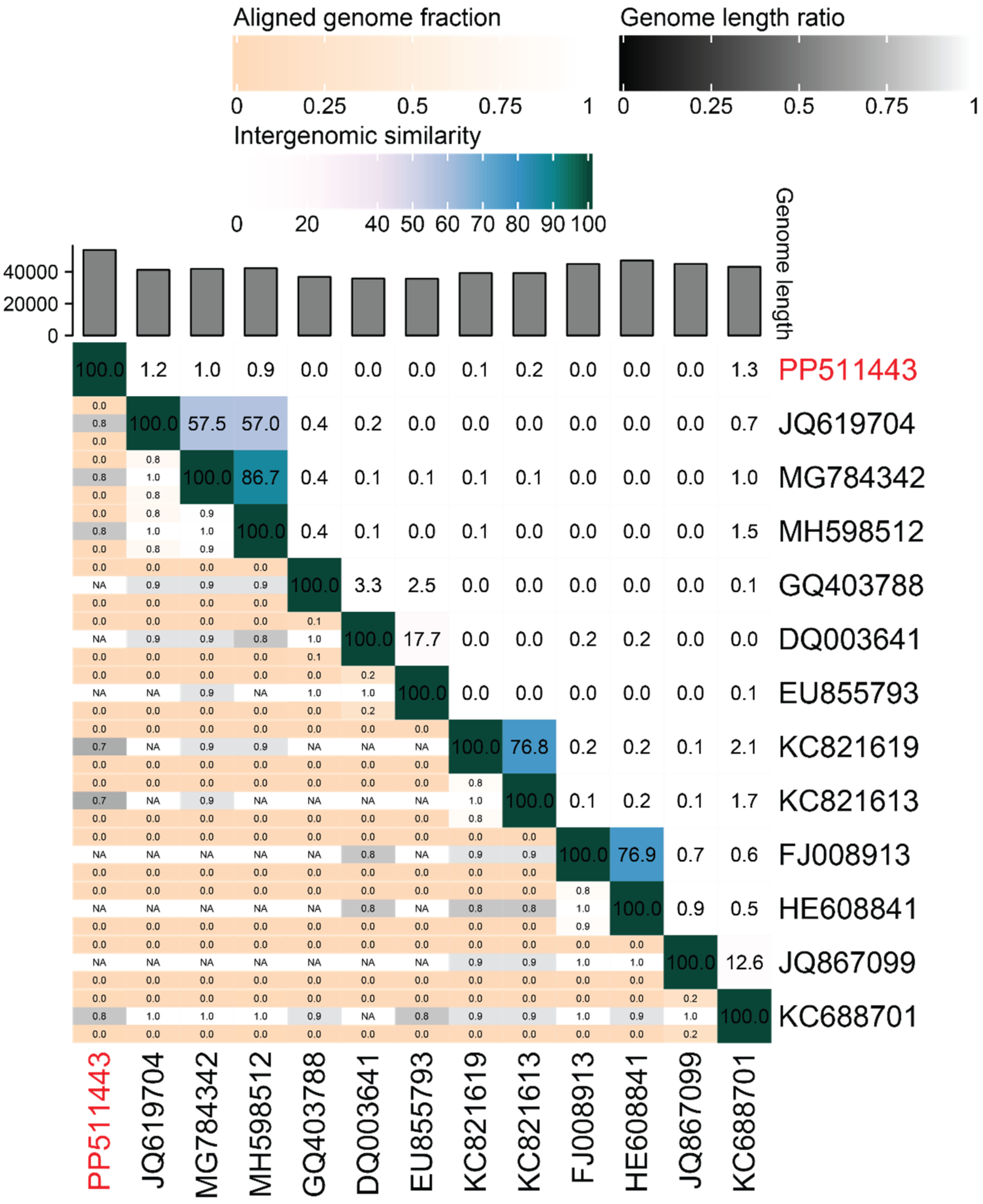

Figure S14. VIRIDIC heatmap from a subset of the caudovirus phylogeny from Figure 18J.

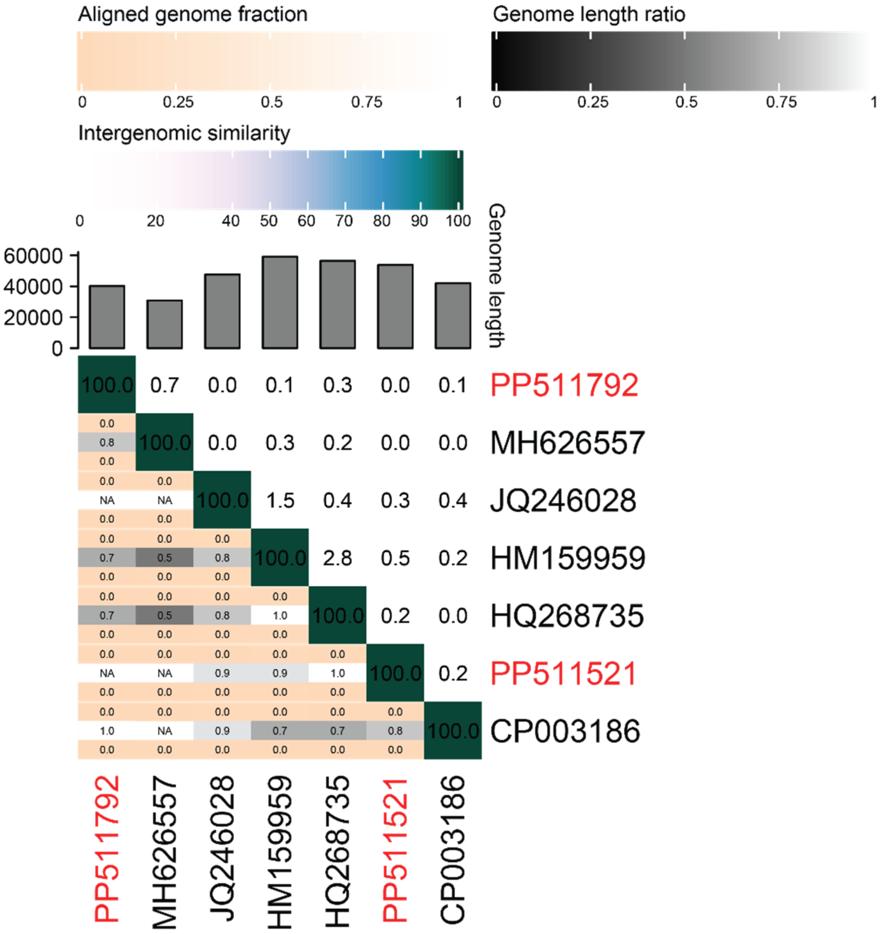

Figure S15. VIRIDIC heatmap from a subset of the caudovirus phylogeny from Figure 18K.
